# Effect of peer counselling on acceptance of modern contraceptives among female refugee adolescents in northern Uganda: A randomised controlled trial

**DOI:** 10.1371/journal.pone.0256479

**Published:** 2021-09-02

**Authors:** Ritah Bakesiima, Jolly Beyeza-Kashesya, James K. Tumwine, Rose Nabirye Chalo, Kristina Gemzell-Danielsson, Amanda Cleeve, Elin C. Larsson

**Affiliations:** 1 Department of Obstetrics and Gynaecology, School of Medicine, College of Health Sciences, Makerere University, Kampala, Uganda; 2 Department of Women’s and Children’s Health, Division of Obstetrics and Gynaecology, Karolinska Institutet, Stockholm, Sweden; 3 Department of Obstetrics and Gynaecology, Mulago Specialised Women and Neonatal Hospital, Kampala, Uganda; 4 Department of Paediatrics and Child Health, School of Medicine, College of Health Sciences, Makerere University, Kampala, Uganda; 5 Department of Nursing, School of Health Sciences, College of Health Sciences, Makerere University, Kampala, Uganda; 6 The WHO Collaborating Centre, Division of Women’s Health, Karolinska University Hospital, Stockholm, Sweden; 7 Department of Women´s Health, South General Hospital, Stockholm, Sweden; 8 Department of Global Public Health, Karolinska Institutet, Stockholm, Sweden; University of North Carolina at Chapel Hill School of Medicine, UNITED STATES

## Abstract

**Background:**

The unmet need for contraceptives among refugee adolescents is high globally, leaving girls vulnerable to unintended pregnancies. Lack of knowledge and fear of side effects are the most reported reasons for non-use of contraceptives amongst refugee adolescents. Peer counselling, the use of trained adolescents to offer contraceptive counselling to fellow peers, has showed effectiveness in increasing use of contraceptives in non-refugee adolescent resarch.

**Objective:**

To determine the effect of peer counselling on acceptance of modern contraceptives among female refugee adolescents in northern Uganda.

**Methods:**

A randomised controlled trial carried out in Palabek refugee settlement in northern Uganda, May to July 2019. Adolescents were included if they were sexually active or in any form of union, wanted to delay child bearing, and were not using any contraceptives. A total of 588 consenting adolescents were randomised to either peer counselling or routine counselling, the standard of care.

**Results:**

Adolescents who received peer counselling were more likely to accept a contraceptive method compared to those who received routine counselling (PR: 1·24, 95% CI: 1·03 to 1·50, p = 0·023). Adolescents whose partners had attained up to tertiary education were more likely to accept a method than those whose partners had secondary or less education (PR: 1·45, 95% CI: 1·02 to 2·06, p = 0·037). In both groups, the most frequently accepted methods were the injectable and implant, with the commonest reasons for non-acceptance of contraception being fear of side effects and partner prohibition.

**Conclusion:**

Our data indicates that peer counselling has a positive effect on same day acceptance of modern contraceptives and should therefore be considered in future efforts to prevent adolescent pregnancies in refugee settings. Future peer counselling interventions should focus on how to effectively address adolescents’ fear of side effects and partner prohibition, as these factors continue to impede decision making for contraceptive uptake.

## Introduction

The unmet need for contraception remains high globally, with the largest numbers among adolescents, migrants, urban slum dwellers and refugees [1]. Globally, about 13 million female adolescents who are sexually active or in union, report a wish to delay childbearing, but are not using any contraceptives [2], illuminating a large unmet need for contraception in this population. Among female adolescents in humanitarian settings, the unmet need for contraception is reported to be more than 30% [3–5] and the contraceptive prevalence is low. The most frequently reported reasons for non-use of contraception among refugee adolescents are poor access to family planning services, fear of side effects, social acceptability (including partner’s approval) and lack of adequate knowledge or information on contraceptives, which are similar for adolescents in non-humanitarian settings [6–8].

An estimated 10 million unintended pregnancies are reported among girls aged 15–17 years in low income countries annually [9]. The numbers of unintended pregnancies are estimated to be even higher among refugee adolescents because they are at a high risk of sexual and/or gender-based violence, abuse, and forced marriages, which may lead to unintended pregnancy [10] and unsafe abortion [11].

Globally, approximately 5.6 million abortions occur each year among adolescents aged 15–19 years [12], 70% of which are unsafe and may result into maternal mortality, morbidity and other health problems [9, 13]. Furthermore, adolescent pregnancy and child birth is associated with many complications and represents the primary cause of death among adolescents 15 to 19 years of age, globally [9]. The maternal complications include obstetric fistula, pregnancy induced hypertension, puerperal sepsis, post-partum depression and other systemic infections [9, 14–17], while the neonatal complications include low birth weight, preterm delivery and other neonatal complications [18–20]. It is therefore of great importance to identify interventions that effectively curb the rate of unintended adolescent pregnancies and associated complications, which in the long run will help improve the livelihood of adolescents.

WHO advocates for contraceptive counselling as one of the main interventions to prevent adolescent pregnancy thereby increasing contraceptive knowledge, dispelling misconceptions and dealing with fear of side effects [21]. A number of studies aiming to increase uptake of contraception among adolescents, both in low and high income settings have focused on peer education/counselling [22–25]. Existing evidence on child development shows that peers become of great significance while adults lose some of their significance in adolescent years [26, 27]. Therefore, the hypothesis of peers in contraceptive counselling is that it should have a positive effect on contraceptive uptake and use. However, previous studies have shown conflicting results [22–25]. The aim of this study was to determine the effect of peer counselling, compared to routine counselling, on same day acceptance of modern contraceptives among female refugee adolescents in northern Uganda.

## Methods

### Trial design and setting

This was a randomised, controlled, single blind, superiority trial with two-parallel groups in a 1:1 allocation ratio, carried out from Palabek refugee settlement in northern Uganda, May to July 2019. Palabek refugee settlement is the one of the newest refugee settlements in Uganda, established in April 2017, and hosting over 53,000 refugees from South Sudan, 85% of whom are women and children [28]. This settlement is faced with a high burden of adolescent pregnancy, especially among girls aged 17–19 years [6]. It has 11 primary and secondary schools, all of which offer free education with a few sessions on sexual and reproductive health and rights. Furthermore, the settlement has three health centres within a 45 minutes’ walk, all of which provide contraceptive services free of charge to all individuals, including adolescents. Contraceptive counselling usually takes place on the health facilities’ family planning day, which occurs once a week, either in a health facility or during the regular outreach activities in the communities. Counselling is provided by a trained nurse and is given in a single group of girls and women seeking counselling that day. Contraceptives are made available to all who need them with stock-outs occurring infrequently due to continued partnerships with organisations like UNFPA and UNHCR, together with the local government which ensure continued supply of the contraceptives.

### Participants

The study included female refugees aged 15 to 19 years who; 1) were sexually active (having had sexual relations within the past three months) or were in any form of union, 2) wanted to delay child-bearing, 3) were currently not using any modern contraceptive method, 4) were residing within Palabek refugee settlement during the study period, and 5) consented to participate in the study. Participants were excluded if they could not comprehend the languages English, Acholi and Arabic (the most commonly used languages in the settlement), or if they were mentally or physically unable to adhere to study procedures like consenting or interviews.

### Intervention

The intervention in this trial was peer counselling, where trained adolescents provided contraceptive counselling to fellow peers with the aid of a standardised World Health Organisation (WHO) contraceptive counselling guide, i.e. the same tool as is being used ny health professionals [29]. Adolescents in the intervention arm received individual peer counselling conducted within their communities, in privacy at the community meeting place. Peer counsellors discussed the advantages and disadvantages of the different contraceptive methods, their safety and effectiveness. They also responded to questions, and dispelled any misconceptions and myths that were brought up by the adolescents. Peers used physical samples of the different contraceptive methods during counselling in order to explain how the methods are used/taken and to demonstrate how implants and intra-uterine devices (IUDs) are inserted. The counselling sessions took approximately 15 to 20 minutes.

Peer counsellors were three female adolescents who were selected from within the settlement and trained to offer contraceptive counselling. They were selected on the basis of their social and sexual maturity, ability to interact verbally, leadership skills, and age. The peer counsellors were aged 16, 18 and 19 years and were given training sessions for four hours daily during five days in conversational, interaction and observational skills, decision making, counselling, confidentiality, problem solving and contraceptive counselling. The training sessions were conducted by a professional family planning specialist who was working in the family planning clinic of one of the health centres in the settlement, together with RB, who gave them training on work ethics, and introduced them to the standard counselling guide.

#### Control

The control group received routine contraceptive counselling, the standard of care within the refugee settlement. This counselling is done in accordance with the family planning guide developed and approved by the Ministry of Health, Uganda, in line with the WHO contraceptive counselling guide [29]. For this study, the routine counselling was done as outreach visits in the communities, conducted by the health centre nurses, which is part of routine care. Each counselling session took about 20 to 30 minutes. Two nurses were selected from the family planning clinic at two of the health centres in the camp, with no particular preference except for their availability and acceptance to contribute to the study. The nurses were not trained specifically for this study, but rather instructed to offer contraceptive counselling to adolescents as they routinely do in the health centre.

### Outcomes

The **primary outcome** was same day acceptance of a modern contraceptive method, defined as being willing to start any method immediately after counselling. This outcome was measured as a binary variable.

**Secondary outcomes** included; type of modern contraceptive method chosen, reasons for non-acceptance to use a modern contraceptive method, and change in willingness to use a method.

Five modern contraceptive methods were made available for this study and these were condoms, oral contraceptives (commonly known as pills), injectable contraceptives, implants and the copper intra-uterine device (IUD).

The change in willingness to use a contraceptive method following counselling was measured as i) positive change—defined as a change from “unwilling” to “willing” to accept a method, ii) negative change—defined as change from “willing” to “unwilling” to accept a method, and iii) no change.

### Sample size

The sample size for this study was estimated using the Hayes and Bennett formula for determining the sample size for an RCT [30]. We used the proportion of contraceptive uptake among adolescents on standard counselling of 0.204 according to a study conducted in Ghana [31]. Assuming a clinically meaningful difference of 10% between the two proportions, the proportion of uptake among peer counselled adolescents was estimated at 0.304, with a 95% level of confidence and power of 80%. When incorporated into the formula, this gave us a sample size of 588, with 294 in each group. Therefore, a total of 588 female refugee adolescents who met the inclusion criteria and consented to participate in the study were randomised to either peer counselling or routine counselling.

### Randomisation

Participants were randomly assigned to either the control or intervention group in a 1:1 allocation ratio using simple randomisation. A random number generator was used to obtain 294 unique and random numbers for two groups. The allocations were not disclosed to anyone. The person who generated the randomisation sequence (RB) did not participate in the execution of the randomisation. Allocation concealment was achieved by enclosing assignments in sequentially numbered opaque sealed envelopes (SNOSE) to ensure that the person randomising (AO) did not know what the next treatment allocation was. In addition, central randomisation (using a randomisation point remote from the trials location) was used within the community.

#### Blinding

Assessment of the outcome among the participants was conducted by a research assistant (SA) who was blinded to the allocation arm of the participant. Due to the nature of the intervention, neither the participants nor the investigators could be blinded. However, they were instructed not to disclose the allocation status to the outcome assessors. In addition, data was entered by VN who had no knowledge of the random allocation.

### Data collection

The refugee settlement is arranged in zones, which are further divided into blocks, each with a leader. Each zone has a meeting place where community meetings are held. Block leaders normally move around to invite people within their blocks for meetings whenever need arises. Data was collected in the community meeting place, where all adolescents within a given area of residence in blocks close to each other had been called upon to converge by block leaders. About 20–30 participants were enrolled in each area per day during the three months of data collection. At the meeting place, participants were informed about the study by the research assistants, and those who were interested were individually and privately screened for eligibility, and individual written informed consent was obtained. Baseline data were then collected by two trained interviewers with the help of an interviewer administered questionnaire to obtain information on social demographics, sexual and reproductive history, partner’s characteristics, and knowledge and previous use of modern contraceptives. All the questionnaires used had been pre-tested and piloted by RB together with the research assistants. The pilot was done among 30 adolescents in a different block of the refugee settlement that was not included in the trial. No adjustments were needed on the final questionnaire.

After obtaining baseline information, participants were sent to the randomisation point where they were randomised and then directed to an allocated room for contraceptive counselling. Following the contraceptive counselling, participants were sent to a trained outcome assessor in a different room. The outcome assessor used a questionnaire to obtain information on both the primary and secondary outcomes of the trial. All interviews took place in a private and enclosed space where conversations could not be overheard. Those who accepted to initiate methods like the condom and oral contraceptive received them immediately while those who had accepted to initiate methods like the injectable, implant and IUD were that same day escorted by the research assistant to the nearest health centre where they received their desired method. All the contraceptive methods were made available by the funders of the study in all the health centres at the time of the study, although the health centres too had their own stock.

### Statistical analysis

STATA version 13.0 was used for data analysis, where continuous variables were summarised as means and standard deviations if normally distributed, and as medians and ranges if skewed, while categorical variables were summarised as proportions and percentages.

Same day acceptance of a modern contraceptive method was analysed as a categorical variable, with acceptance of a method coded as “1” and non-acceptance coded as “0”. Chi-square test was used to compare same day acceptance between the two study groups. Other factors influencing contraceptive acceptance were estimated using the modified poisson regression model. This model was considered the most appropriate method because of the high prevalence of acceptance observed (>30%) in the study population. Bivariate analysis was done by fitting a model for all the independent variables with the outcome. All the variables that gave a p-value ≤0.2 at the bivariate analysis were considered for multivariate analysis. Also considered for the multivariate analysis were variables that were known to be plausible from previous research.

All the variables which fulfilled the criteria for multivariate analysis were run in a stepwise model in which variables were dropped according to their significance. Only those with p-value less than 0.05 were retained. Two-way product terms were then formed among the variables which had been retained after the stepwise model. These product terms were used to assess for interaction. Confounding was then assessed for and a variable was considered a confounder if it caused a greater than or equal to 10% change in the prevalence ratio of the primary outcome [32]. Prevalence ratios along with their 95% confidence intervals were reported, and statistical significance reported at p<0.05.

### Ethical considerations

This study obtained ethical approval from the Makerere University School of Medicine Higher Degrees Research Ethics Committee (REC REF 2018–059), and from Uganda National Council of Science and Technology (UNCST). We further sought administrative clearance from the Office of the Prime minister, Department of Refugees, which offers researchers permission to carry out research among refugees. Before inclusion into the study, written informed consent was obtained from all participants. Prior to this, all participants had been given written and oral information about the study, that their participation was voluntary, that they could withdraw from the study at any timepoint and that their answers would be kept anonymous. We ensured that participant confidentiality was maintained by the use of unique number codes instead of participants’ names and by conducting counselling in a private place where the conversations could not be over heard. For this study, there was no need to obtain parental consent for participants below 18 years because the National Policy Guidelines and Service Standards for Sexual and Reproductive Health and Rights state that all individuals who are sexually active are eligible for family planning services irrespective of age provided that they have been educated and counselled, with no verbal or written consent required from the parent, guardian or spouse before receiving the family planning service [33].

This study is registered with Pan African clinical trial registry, number PACTR201808666856363.

## Results

### Participant flow

**Fig 1:**
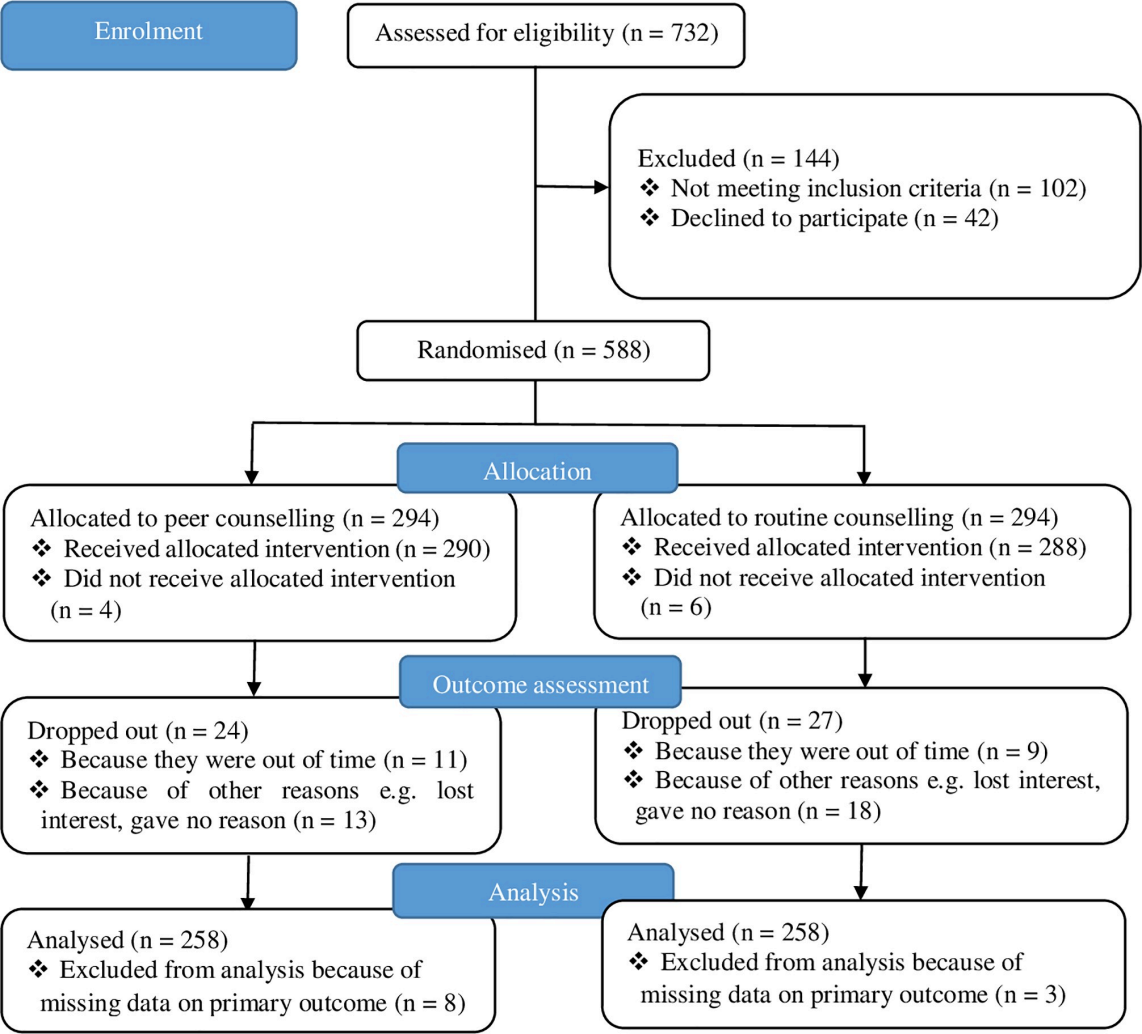
Trial profile showing enrolment and randomisation of study participants.

A total of 732 female refugee adolescents were assessed for eligibility to participate in the study from May to July 2019. Amongst these, 102 did not meet the inclusion criteria, while 42 declined to participate. Therefore 588 participants remained who were randomised to either intervention or control with 294 in each arm. Some participants (n = 10) did not receive the allocated counselling, while 51 dropped out before outcome assessment. A total of 516 were included in the analysis (Fig 1).

### Background characteristics of the study participants

In terms of the social demographics, most of the participants were to 18 and 19 years, had attained up to primary as their highest level of education, were unemployed and cohabiting. Regarding the sexual and reproductive health characteristics, most of the participants had had their first sex by 16 years, had ever been pregnant, and had at least one child alive (Table 1).

**Table 1:** Background characteristics of the study participants.

Variable	Peer counselling (n = 258)	Routine counselling (n = 258)
**Age** (Mean, SD)	18.4, 0.83	18.4, 0.88
**Age categorised**		
15 to 17	35 (13.6)	42 (16.3)
18 to 19	223 (86.4)	216 (83.7)
**Religion**		
Catholic	132 (51.1)	133 (51.6)
Anglican	64 (24.8)	54 (20.9)
Adventist	32 (12.4)	24 (9.3)
Other (Pentecostal, EFC, AIC)	30 (11.6)	47 (18.2)
**Ethnicity**		
Acholi	213 (82.6)	192 (74.4)
Nuer	2 (0.8)	7 (2.7)
Dinka	7 (2.7)	8 (3.1)
Lotuho	12 (4.6)	17 (6.6)
Other (Shilluk, Luo, Bari)	24 (9.3)	34 (13.2)
**Education**		
None	18 (7.0)	33 (12.8)
Primary	194 (75.2)	167 (64.7)
Secondary	40 (15.5)	54 (20.9)
Tertiary	6 (2.3)	4 (1.6)
**Occupation**		
Unemployed	109 (42.2)	96 (37.2)
Employed/Self employed	7 (2.7)	11 (4.3)
Peasant farmer	82 (31.8)	90 (34.9)
Student	60 (23.3)	61 (23.6)
**Marital status**		
Single	81 (31.4)	84 (32.6)
Cohabiting	114 (44.2)	104 (40.2)
Married	46 (17.8)	51 (19.8)
Separated/Divorced/Widowed	17 (6.6)	19 (7.4)
**Age at first sex** (Mean, SD)	16.3, 1.08	16.4, 1.05
**Ever been pregnant**		
Yes	170 (65.9)	167 (64.7)
No	88 (34.1)	91 (35.3)
Number of children alive* (Mean, SD)	1.62, 0.78	1.43, 0.66
**Age of partner** *		
16 to 25	89 (46.1)	82 (45.6)
26 to 35	98 (50.8)	85 (47.2)
36 to 60	6 (3.1)	13 (7.2)
**Partner’s education** *		
None	4 (2.1)	3 (1.7)
Primary	52 (26.9)	65 (35.9)
Secondary	133 (68.9)	105 (58.0)
Tertiary	4 (2.1)	8 (4.4)
**Partner’s occupation** *		
Employed/Self employed	49 (25.4)	52 (28.6)
Unemployed	74 (38.3)	84 (46.1)
Peasant farmer	50 (25.9)	32 (17.6)
Student	20 (10.4)	14 (7.7)
**Ever used modern contraceptives**		
Yes	44 (17.1)	28 (10.9)
No	214 (82.9)	230 (89.1)

EFC–Evangelical Free Church

AIC–African Initiated Church

*—value taken from only those with partners or only those who had ever been pregnant

### Study outcomes in the different study groups

Results relating to the primary and secondary outcomes are presented in Table 2 and presented by study group. The outcomes *same day acceptance* and *reasons for non-acceptance* differed between study groups i.e participants who received peer counselling had more acceptors (58.5%) than those who received routine counselling (49.6%). The commonest reason for non-acceptance in the peer counselling group was lack of time contrary to fear of side effects in the routine counselling group. There was no difference in the type of contraceptive method accepted between the two study groups (Table 2).

**Table 2:** Primary and secondary outcomes by study group.

Outcome	N	Peer counselling	Routine counselling	p-value
**Same day acceptance of a modern contraceptive**				
Yes	279	151 (58.5)	128 (49.6)	
No	237	107 (41.5)	130 (50.4)	0.013
**Type of modern contraceptive method chosen**				
Condoms	20	11 (7.3)	9 (7.0)	
Oral contraceptive (pill)	42	23 (15.2)	19 (14.8)	
Injectable contraceptive	129	73 (48.4)	56 (43.8)	
Implant	86	44 (29.1)	42 (32.8)	
IUD	2	0 (0.0)	2 (1.6)	0.649
**Change in willingness to accept a contraceptive method**				
Positive change	112	55 (21.3)	57 (22.1)	
Negative change	15	7 (2.7)	8 (3.1)	
No change	389	196 (76.0)	193 (74.8)	0.933
**Reasons for non-acceptance**				
Infrequent sex	3	2 (1.9)	1 (0.7)	
Cultural/religious prohibition	43	17 (15.9)	26 (20.0)	
Partner prohibition	52	29 (27.1)	23 (17.7)	
Fear of side effects	81	25 (23.4)	56 (43.1)	
Lack of knowledge	9	1 (0.9)	8 (6.2)	
No time to go to health centre	49	33 (30.8)	16 (12.3)	<0.001

### Bivariate analysis of participants’ background characteristics and same day acceptance of modern contraceptives

Variables associated with same day acceptance at bivariate analysis were study group, ethnicity, occupation, marital status, partner’s age, partner’s education and partner’s occupation, and preveious use of modern contracceptives. Participants who were married were more likely to accept a method compared to those who were single or cohabiting, while those with older partners were less likely to accept a contraceptive method as shown in Table 3.

**Table 3:** Bivariate analysis.

Variable	N	Acceptance	Non-acceptance	Prevalence Ratio (95% CI)	p-value
**Type of counselling**					
Routine counselling	258	128 (49.6)	130 (50.4)	1.00	
Peer counselling	258	151 (58.5)	107 (41.5)	1.18 (1.00 to 1.38)	**0.043**
**Age categorised**					
15 to 17	77	41 (53.2)	36 (46.8)	0.98 (0.78 to 1.23)	0.876
18 to 19	439	238 (54.2)	201 (45.8)	1.00	
**Religion**					
Catholic	265	145 (54.7)	120 (45.3)	1.00	
Anglican	118	62 (52.5)	56 (47.5)	0.96 (0.78 to 1.18)	0.696
Adventist	56	27 (48.2)	29 (51.8)	0.88 (0.66 to 1.18)	0.397
Other (Pentecostal, EFC, AIC)	77	45 (58.4)	32 (41.6)	1.07 (0.86 to 1.33)	0.554
**Ethnicity**					
Acholi	405	229 (56.5)	176 (43.5)	1.00	
Nuer	9	2 (22.2)	7 (77.8)	0.39 (0.12 to 1.34)	**0.136**
Dinka	15	5 (33.3)	10 (66.7)	0.59 (0.29 to 1.21)	**0.151**
Lotuho	29	15 (51.7)	14 (48.3)	0.92 (0.64 to 1.31)	0.630
Other (Shilluk, Luo, Bari)	58	28 (48.3)	30 (51.7)	0.85 (0.65 to 1.13)	0.269
**Occupation**					
Unemployed	205	100 (48.8)	105 (51.2)	1.00	
Employed/Self employed	18	12 (66.7)	6 (33.3)	1.37 (0.96 to 1.95)	0.085
Peasant farmer	172	106 (61.6)	66 (38.4)	1.26 (1.05 to 1.52)	**0.012**
Student	121	61 (50.4)	60 (49.6)	1.03 (0.82 to 1.30)	0.775
**Marital status**					
Single	165	83 (50.3)	82 (49.7)	1.00	
Cohabiting	97	51 (52.6)	46 (47.4)	1.05 (0.82 to 1.33)	0.721
Married	218	127 (58.3)	91 (41.7)	1.16 (0.96 to 1.40)	**0.128**
Separated/Divorced/Widowed	36	18 (50.0)	18 (50.0)	0.99 (0.69 to 1.43)	0.974
**Age at first sex** (Mean, SD)					
12 to 15	104	57 (54.8)	47 (45.2)	1.00	
16 to 17	336	178 (53.0)	158 (47.0)	0.97 (0.79 to 1.18)	0.741
18 to 19	76	44 (57.9)	32 (42.1)	1.06 (0.81 to 1.37)	0.679
**Ever been pregnant**					
No	179	92 (51.4)	87 (48.6)	1.00	
Yes	337	187 (55.5)	150 (45.5)	1.08 (0.91 to 1.28)	0.382
Age of partner* (Mean, SD)		26.4 (5.17)	27.3 (5.15)	0.98 (0.96 to 1.01)	**0.139**
16 to 25	171	99 (57.9)	72 (42.1)	1.00	
26 to 35	183	95 (51.9)	88 (48.1)	0.90 (0.74 to 1.08)	0.259
36 to 60	19	12 (63.2)	7 (36.8)	1.09 (0.76 to 1.57)	0.642
Partner’s education*					
None	7	5 (71.4)	2 (28.6)	1.35 (0.82 to 2.22)	0.241
Primary	117	62 (53.0)	55 (47.0)	1.00	
Secondary	238	130 (54.6)	108 (45.4)	1.03 (0.84 to 1.27)	0.774
Tertiary	12	9 (75.0)	3 (25.0)	1.42 (0.98 to 2.05)	**0.065**
Partner’s occupation*					
Employed/Self employed	101	50 (49.5)	51 (50.5)	1.00	
Unemployed	158	87 (55.1)	71 (44.9)	1.11 (0.87 to 1.42)	0.390
Peasant farmer	82	52 (63.4)	30 (36.6)	1.28 (0.99 to 1.66)	**0.059**
Student	34	18 (52.9)	16 (47.1)	1.07 (0.74 to 1.55)	0.725
**Ever used modern contraceptives**					
No	444	233 (52.5)	211 (47.5)	1.00	
Yes	72	46 (63.9)	26 (36.1)	1.23 (1.01 to 1.49)	**0.037**

### Multivariate analysis of the factors associated with same day acceptance of a modern contraceptive

Variables with a p-value less than 0.2 at bivariate analysis were included in the multivariate analysis (Table 4). However, participants’ age was also included in the multivariate analysis despite having a p-value greater than 0.2 at bivariate analysis as it may influence contraceptive acceptance according to previous research [34, 35]. A stepwise (backward) model was run with all these variables, and the only variables that were statistically significant were study group (PR = 1.24, 95% CI: 1.03 to 1.50, p = 0.023) and partner’s education (PR = 1.45, 95% CI: 1.02 to 2.06, p = 0.037 for tertiary education, PR = 1.01, 95% CI: 0.82 to 1.23, p = 0.958 for secondary education, and PR = 1.31, 95% CI: 0.80 to 2.16, p = 0.286 for no education). These variables were assessed for interaction between each other and for confounding with the other independent variables that were included in the multivariate model. However, no interaction or confounding was found.

**Table 4:** Multivariate analysis of the factors associated with same day acceptance to use modern contraceptives.

Variable	Prevalence Ratio	95% CI	P-Value
**Type of counselling**			
Routine counselling	1.00		
Peer counselling	1.24	1.03 to 1.50	0.023
**Partner’s education**			
None	1.31	0.80 to 2.16	0.286
Primary	1.00		
Secondary	1.01	0.82 to 1.23	0.958
Tertiary	1.45	1.02 to 2.06	0.037

## Discussion

This study provides evidence on the effect of peer counselling on same day acceptance of modern contraceptives among female refugee adolescents in northern Uganda. The results show that adolescents who received peer counselling were 24% more likely to accept a modern contraceptive method compared to those who received routine counselling. These findings are in line with previous work on peer counselling in Turkey which reported an increase in use of contraceptive services by 37.5% [23] but in contradiction to results from a trial conducted in the US which reported no significant association between peer counselling and same-day desire for LARC [22]. the latter trial looked at acceptance of LARCs only whereas our study looked at acceptance of any modern contraceptive which could explain differences in results. Previous studies have shown that adolescents assign greater priority to and easily adopt behaviours and norms of their fellow peers [26, 27] and that adolescents are more easily affected by peer influence compared to children and adults [36]. Our trial supports these findings and indicates that peer counselling may be an important tool in the quest to reduce unintended pregnancies among refugee adolscents.

The most frequently reported reason for non-acceptance in the routine counselling group was fear of side effects, which was reported almost twice as high in this group (43.1%) compared to the peer counselling group (23.4%). This could mean that the routine counselling may not have been as effective in dealing with the fear of side effects, compared to peer counselling. Furthermore, receiving information about the method side effects could be more believable when it is coming from a peer. Partner prohibition was the most important cause for non-acceptance in the peer counselling group (27.1%) and the third most important in the routine counselling group (17.7%). One could view this as being due to the fact that peer counselling, in spite of its ability to increase contraceptive acceptance, may not be able to address partner prohibition.

Furthermore, findings from our trial show that partner’s education is of importance to contraceptive acceptance, with higher education being associated with a higher degree of uptake. This is in line with findings from previous research in Nepal which reported that men with secondary education or higher were more likely to rely on certain contraceptive choices than their counterparts with less education [37]. This can be explained by a finding that with increase in male partner’s education, there is an increase in support offered to the spouse to use contraceptives [38]. Involving partners in contraceptive counselling has been identified as an effective strategy to address partner prohibition [39]. This strategy could be considered in future peer counselling interventions in order to address partner prohibition and encourage partner’s support to contraceptive use also among partners with lower education.

The strengths of this study are: i) the design, using a randomised controlled trial which provides data to make causal inferences with the strongest form of empirical evidence. However, it is important to note that the confidence interval for the effect of the intervention was wide (1.03 to 1.50), so this should be treated with caution; ii) the outcome assessor was blinded which further helps to minimise performance/assessment bias; iii) the outcome was assessed on the same day of the intervention which helps to minimise contamination that could have occurred with participant mixing if not assessed on the same day; iv) all the questionnaires had been piloted and pretested to ensure that meaning was not altered hence minimising misclassification bias.

This study was not without limitations. Some participants were not included in the analysis due to missing data on the primary outcome. However, the characteristics of the excluded participants did not differ much from those of the participants who were included in the analysis. Questionnaires were used to obtain information on participant characteristics which may be subject to social desirability bias. This was however minimised by conducting interviews in private, enclosed and friendly environments. Furthermore, since the outcome was assessed immediately after counselling, the participants may not have been given enough time to think about their decision. It is possible that many of those who said no could have picked interest in using contraceptives if they had been given more time to think about it. Lastly, a number of participants dropped out before outcome assessment and this could have reduced the power of the study to detect some associations which could have otherwise been present.

## Conclusion

We found that adolescents who received peer counselling were more likely to accept modern contraceptives compared to those who received routine counselling, indicating that peer counselling has a positive effect on acceptance of modern contraceptives and should therefore be considered in future efforts to prevent adolescent pregnancies in refugee settings. The intervention also reduced the poroprtion of women mentioning side-effects as a reason to declining contraception.

However, further studies are needed to examine the feasibility of scaling up this intervention, and its cost-effectiveness. The importance of partner support in relation to contraceptive decision-making warrants more attention and strengthen refugee girls`autonomy so as to enable them to avoid unintended pregnancy and early childbearing.

## Supporting information

S1 Checklist CONSORT checklist. (DOC)

S1 File Data for the study. (XLS)

S2 File Study protocol. (DOCX)
