# Supplementary material for: Effect of peer counselling on acceptance of modern contraceptives among female refugee adolescents in northern Uganda: A randomised controlled trial
Source: PLoS One. 2021 Sep 2;16(9):e0256479. doi: 10.1371/journal.pone.0256479 (PMC8412258; doi:10.1371/journal.pone.0256479)
Supplement: S2 File — (DOCX) [file pone.0256479.s003.docx]

**MAKERERE UNIVERSITY**

**UPTAKE OF MODERN CONTRACEPTIVES AMONG REFUGEE ADOLESCENTS IN NORTHERN UGANDA: DETERMINANTS, ADHERENCE, LIVED EXPERIENCES AND EFFECT OF PEER COUNSELLING**

**BY**

**BAKESIIMA RITAH (BLT, MSc.)**

**SUPERVISORS**

**DR. JOLLY BEYEZA-KASHESYA (MBChB, MMed OBS/GYN, PhD)**

**DR. ROSE CHALO NABIRYE (RN, BSN, MPH, PhD)**

**DR. ELIN LARSSON (MSc, PhD)**

**PROF. KRISTINA GEMZELL-DANIELSSON (MMed, PhD)**

**A PROPOSAL SUBMITTED TO MAKERERE UNIVERSITY IN PARTIAL FULFILLMENT OF THE REQUIREMENTS FOR THE AWARD OF DOCTOR OF PHILLOSOPHY IN REPRODUCTIVE HEALTH**

**SEPTEMBER 2018**

**DECLARATION**

I, **Ritah Bakesiima,** hereby declare that the work submitted in this proposal is my own original compilation and has not been submitted to any other institution of higher learning for any award of any academic qualification. All this work is original unless otherwise stated.

Signature: ..................................................... Date: ............................................

This dissertation has been submitted for examination with the approval of my supervisors:

1. Dr. Jolly Beyeza-Kashesya

Department of Obstetrics and Gynaecology, School of Medicine

College of Health Sciences, Makerere University

Signature: ..................................... Date: ............................................

1. Dr. Rose Chalo Nabirye

Department of Nursing, School of Health Sciences

College of Health Sciences, Makerere University

Signature: .................................... Date: ............................................

1. Dr. Elin Larsson

Department of Women and Children’s Health

Karolinska Institutet

Signature: ..................................... Date: ............................................

1. Prof. Kristina Gemzell-Danielsson

Department of Women and Children’s Health

Karolinska Institutet

Signature: .................................... Date: .........................................

**TABLE OF CONTENTS**

Contents

[LIST OF ACRONYMS iii](#_Toc515715534)

[OPERATIONAL DEFINITIONS iv](#_Toc515715535)

[**CHAPTER ONE** **1**](#_Toc515715536)

[**INTRODUCTION** **1**](#_Toc515715537)

[1.1 Background 1](#_Toc515715538)

[1.2 Problem Statement 2](#_Toc515715539)

[1.3 Justification 4](#_Toc515715540)

[1.4 Research Questions 5](#_Toc515715541)

[1.5 General Objective 5](#_Toc515715542)

[1.6 Specific objectives 5](#_Toc515715543)

[1.7 Conceptual and theoretical framework 6](#_Toc515715544)

[1.7.1 Conceptual framework 6](#_Toc515715545)

[1.7.2 Theoretical framework 8](#_Toc515715546)

[**CHAPTER TWO** **9**](#_Toc515715548)

[**LITERATURE REVIEW** **9**](#_Toc515715549)

[2.1 Modern contraceptives 9](#_Toc515715550)

[2.2 Modern contraceptive use among refugee adolescents 11](#_Toc515715551)

[2.2.1 Factors associated with modern contraceptive use among refugee adolescents 12](#_Toc515715552)

[2.3 Adherence to modern contraceptives among refugee adolescents 13](#_Toc515715553)

[2.3.1 Factors associated with adherence to modern contraceptives among refugee adolescents 14](#_Toc515715554)

[2.4 Effect of peer counselling on uptake of modern contraceptives 15](#_Toc515715555)

[2.5 Lived experiences of refugee adolescents on modern contraceptives 16](#_Toc515715556)

[**CHAPTER THREE** **18**](#_Toc515715557)

[**METHODS** **18**](#_Toc515715558)

[3.1 Study setting 18](#_Toc515715559)

[3.2 Study design 18](#_Toc515715560)

[3.3 Sub study 1 20](#_Toc515715561)

[3.3.1 Objective: 21](#_Toc515715562)

[3.3.2 Study design: 21](#_Toc515715563)

[3.3.3 Population: 21](#_Toc515715564)

[3.3.4 Eligibility criteria: 21](#_Toc515715565)

[3.3.5 Sample size: 21](#_Toc515715566)

[3.3.6 Sampling procedure: 23](#_Toc515715567)

[3.3.7 Data collection: 23](#_Toc515715568)

[3.3.8 Data analysis 24](#_Toc515715569)

[3.4 Sub-study II: 24](#_Toc515715570)

[3.4.1 Objective: 24](#_Toc515715571)

[3.4.2 Study design: 25](#_Toc515715572)

[3.4.3 Hypothesis: 25](#_Toc515715573)

[3.4.4 Study population: 25](#_Toc515715574)

[3.4.5 Eligibility criteria: 25](#_Toc515715575)

[3.4.6 Sample size: 26](#_Toc515715576)

[3.4.7 Sampling procedure: 26](#_Toc515715577)

[3.4.8 Intervention: 27](#_Toc515715578)

[3.4.9 Control: 27](#_Toc515715579)

[3.4.10 Data collection: 28](#_Toc515715580)

[3.4.11 Data analysis 28](#_Toc515715581)

[3.5 Sub-study III: 29](#_Toc515715582)

[3.5.1 Objective: 29](#_Toc515715583)

[3.5.2 Study design: 29](#_Toc515715584)

[3.5.3 Population: 29](#_Toc515715585)

[3.5.4 Eligibility criteria: 29](#_Toc515715586)

[3.5.5 Sample size: 30](#_Toc515715587)

[3.5.6 Sampling procedure 31](#_Toc515715588)

[3.5.7 Data collection: 31](#_Toc515715589)

[3.5.8 Follow up strategy: 32](#_Toc515715590)

[3.5.9 Non-adherence scale: 32](#_Toc515715591)

[3.5.10 Data analysis: 32](#_Toc515715592)

[3.6 Sub-study IV: 33](#_Toc515715593)

[3.6.1 Objective: 33](#_Toc515715594)

[3.6.2 Study design: 33](#_Toc515715595)

[3.6.3 Population: 33](#_Toc515715596)

[3.6.4 Sample size: 34](#_Toc515715597)

[3.6.5 Data collection and analysis: 34](#_Toc515715598)

[3.7 Data management 34](#_Toc515715599)

[3.8 Ethical considerations 35](#_Toc515715600)

[**REFERENCES** 36](#_Toc515715601)

# LIST OF ACRONYMS

ARH Adolescent Reproductive Health

CI Confidence Interval

CIC Combined Injectable Contraceptive

COC Combined Oral Contraceptive

DMPA Depot-medroxyprogesterone acetate

IUD Intra-uterine device

LARC Long Acting Reversible Contraceptives

MoH Ministry of Health

POI Progestin Only Injectable

POP Progestin Only Pill

RCT Randomised Controlled Trial

SOPs Standard Operating Procedures

STDs Sexually Transmitted Diseases

UDHS Uganda Demographic Health Survey

UNFPA United Nations Population Fund

UNHCR United Nations High Commissioner for Refugees

UNICEF United Nations International Children's Emergency Fund

WHO World Health Organisation

WRC Women’s Refugee Commission

# OPERATIONAL DEFINITIONS

**Adolescents:** These are persons between the ages of 10 and 19 years (WHO, 2018a).

**Contraceptive uptake:** This will be defined as the acceptance and initiation of contraceptives by someone who has not been previously using them.

**Contraceptive adherence:** The use of a contraceptive method in an ongoing and consistent manner so as to prevent pregnancy (Jay, 1989). For adherence to be achieved, both continuation and correct use of the contraceptives are required.

**Lived experiences:** These are the experiences and choices of a given person obtained from direct, first-hand accounts of that person involved (Given, 2008).

**Peer:** This is a person of the same age, social position and having the same abilities as refugee adolescents.

**Peer counselling:** A process through which trained peers provide knowledge, emotional, social and practical help to fellow peers regarding contraceptive use.

**Modern contraceptives:** Modern contraceptives are birth control or family planning methods that include the pill (oral contraceptive), injectables, implant, intra-uterine device (IUD) and condoms.

# ABSTRACT

**Background:** Pregnancies among refugee adolescents have continued to be on the rise owing to an unrelenting low uptake of modern contraceptives, and a poor adherence among the few adolescents that are using the modern contraceptives. The low uptake and poor adherence have been attributed to limited knowledge on, and fear of side effects of modern contraceptives among the refugee adolescents. Adolescent pregnancies leave the affected girls vulnerable to the complications associated with pregnancy and child birth which include obstetric fistula, hemorrhagic syndrome and pregnancy induced hypertension among others. It is therefore important for us to study interventions that will help increase uptake of modern contraceptives among refugee adolescents with an aim of reducing adolescent pregnancies and their associated complications especially among refugee adolescents.

**Objective:** This study aims at determining the uptake of modern contraceptives among refugee adolescents in Northern Uganda, the associated factors, adherence to the modern contraceptives, effect of peer counselling and to explore the lived experiences of refugee adolescents on modern contraceptives.

**Methods:** Mixed methods will be used in this study. Study I, whose objective is to determine the prevalence and factors associated with modern contraceptive use among refugee adolescents in Northern Uganda will be a cross sectional study, carried out between August and September 2018 involving 835 female adolescents aged 10 to 19 years from Palabek refugee settlement. Study II, whose objective is to determine the effect of peer counselling on uptake of modern contraceptives among refugee adolescents will be a randomised, controlled, two-group, parallel, outcome assessor blinded trial carried out between October and December 2018, involving 588 female adolescents aged 15 to 19 years from Palabek refugee settlement. Study III, whose objective is to determine the rates and predictors of adherence to modern contraceptives among refugee adolescents in Northern Uganda will be a single prospective cohort study, involving 274 female adolescents aged 15 to 19 years who are new acceptors of modern contraceptives. These will be followed up for a period of six months from January to June 2019 from Palabek refugee settlement. Study IV, whose objective is to explore the lived experiences of refugee adolescents on modern contraceptives in Northern Uganda will be phenomenological study carried out between August and October 2019, among purposively obtained female adolescents aged 15 to 19 years who will have used modern contraceptives for at least 6 months. Questionnaires will be used to obtain information on study variables like social demographics, sexual and reproductive history, spousal information and knowledge and use of modern contraceptives. Data will be analysed using STATA version 13.0.

**Utility:** Information obtained from this study will help improve our understanding of the effect of peer counselling on contraceptive uptake and adherence among refugee adolescents in Northern Uganda. This will guide us better in programming and designing strategies to help improve uptake and in the long run, reduce adolescent pregnancies and associated complications among refugee adolescents.

# CHAPTER ONE

# INTRODUCTION

## 1.1 Background

Globally, adolescent pregnancy rates are still high, with an estimated birth rate of 44 births per 1,000 girls aged 15 to 19 years (UNICEF, 2019). In developing countries, approximately 770,000 girls under 15 years of age and about 12 million aged 15 to 19 years are reported to give birth annually (WHO, 2020). The number of adolescent pregnancies would even be much higher if all pregnancies, and not just the births, were included because an estimated 3 million girls between 15 and 19 years undergo unsafe abortions annually (UNFPA, 2017a).

In low and middle income countries, 20,000 girls under the age of 18 give birth daily, which amounts to 7.3 million births annually (UDHS, 2016). This is an estimated 95% of all the births among girls aged 15 to 19 years worldwide, with the highest rates occurring in sub-Saharan Africa (WHO, 2018b). Furthermore, more than half of all unintended adolescent pregnancies in low income countries are terminated (Sedgh *et al*., 2014).

In Uganda particularly, adolescent pregnancies are currently on the rise and were reported to have increased from 24% in 2011 to 25% in 2016 (UDHS, 2016). These increasing rates of adolescent pregnancies are even worse among refugee adolescents ((UNHCR, 2011).

Adolescent pregnancies in Uganda are still on the rise because of low contraceptive use among sexually active adolescents (Kabagenyi *et al*., 2016). which was reported to be less than 15% among refugee adolescents in Uganda (UNHCR, 2011). This low uptake of contraceptives among adolescents has been attributed to a number of reasons such as unpredictable and irregular sexual activity, limited access to contraception, and inadequate knowledge on contraceptives (Blanc *et al*., 2009). These factors could also lead to contraceptive non-adherence and discontinuation among the few adolescents who may be using the contraceptives. A study by Blanc and her colleagues in 2009 reported a contraceptive discontinuation rate of 50.1% among adolescents in Tanzania (Blanc *et al*., 2002) while a study by Boamah et al in Ghana reported a contraceptive adherence rate of 22.9% among adolescents (Boamah *et al*., 2014). This non-adherence and discontinuation if not addressed, can lead to an increase in the rates of adolescent pregnancies. Several other reasons have been reported to lead to contraceptive non-adherence and discontinuation among adolescents. These include poor service quality, commodity stock-outs, contraceptive side effects, availability of a sufficient choice of methods, ineffective referral methods and limited contraceptive counselling among others (FP2020, 2015).

For success in reduction of adolescent pregnancies to be achieved, adolescents need to be availed with contraceptive information, methods and services (Blanc *et al*., 2009). However, the solution has been mentioned to lie in contraceptive counselling / education (WHO, 2007, Dehlendorf *et al*., 2014). Counselling is necessary to provide accurate information on the mechanism of action of the contraceptives, their efficacy as well as safety (WHO, 2007). Kaunitz in 2017 states that the goal of contraceptive counselling is to educate women about contraception, discuss current and future contraceptive needs, and select a method, to avoid the risks of unintended pregnancy (Kaunitz, 2017). A study in Malawi showed that adolescents who had been counselled by trained counsellors were more likely to take up and continue on contraception than those who were not (Lemani *et al*., 2017).

Several studies have reported the benefits of using peer counselling in contraceptive services for adolescents, and enhancement of their adherence to contraception through increased knowledge and positive change in attitudes which are important in contraceptive decision making (Wilson *et al*., 2016). This study therefore aims to assess the prevalence of modern contraceptive use, the effect of peer counselling on contraceptive uptake, adherence and lived experiences among refugee adolescents in Northern Uganda.

## 1.2 Problem Statement

Adolescent pregnancies among refugees are continuously on the rise owing to, among other things, insecurity and low contraceptive uptake (UNHCR, 2017). This has left many of the refugee adolescents vulnerable to complications associated with adolescent pregnancy and child birth which include abortion, obstetric fistula, pregnancy induced hypertension, haemorrhagic syndrome and premature rupture of membranes among others (Azevedo *et al*., 2015).

These complications associated with adolescent pregnancy and childbirth are the leading cause of death among adolescents aged 15 to 19 years globally (WHO, 2018b), and in Uganda, 17.2% of all maternal deaths are among adolescents (UDHS, 2016). In a humanitarian and fragile setting, an estimated 500 women, most of whom are adolescents, die daily from complications associated with pregnancy and child birth (UNHCR, 2016). This is because early child bearing increases the risk for both the mother and the child, since most of these adolescents are not yet physically ready for pregnancy or even child birth and are therefore more vulnerable to complications (UNFPA, 2017a). Furthermore, more than half of the adolescents terminate their pregnancies in illegal and unsafe circumstances and this further increases their mortality and morbidity (Sedgh *et al*., 2014).

Inasmuch as contraception was introduced among adolescents in order to curb the rates of pregnancy and its associated complications, many adolescents are still getting pregnant due to low contraceptive uptake and poor adherence to contraceptives. Some even end up with repeat pregnancies with short birth intervals which affect both the mother and their children. While the mothers may suffer malnutrition, mental health problems such as depression and anxiety, drop out of school and poverty, their children may face developmental and psychosocial delays coupled with growth challenges (Crowne *et al*., 2012, Rutstein, 2005).

Several factors have been mentioned to directly or indirectly affect uptake of contraceptives among refugee adolescents and these include: social demographics like age, religion, tribe and level of education, individual factors like misconceptions, peer influence and fear of side effects, health service related factors like distance to the health facility and attitude of health workers. For example, less than 30% of the young people in Uganda have accurate knowledge about, or can access quality information on contraception according to UNFPA (UNFPA, 2017b). This proportion is even much lower in refugee populations. Furthermore, over 79% of refugee adolescents in Nigeria were reported not to use modern contraceptives because of fear of side effects (Okanlawon *et al*., 2010).

With an aim of addressing the aforementioned challenges, interventions were implemented by UNHCR and UNFPA to improve uptake of contraceptives among adolescents in humanitarian settings which included training of health workers in contraceptive counselling and making contraceptive services more available (WHO, 2017). However, the uptake is still very low so this shows that the interventions have not been effective enough in increasing contraceptive uptake among refugee adolescents.

Therefore, understanding the drivers of low uptake of contraceptives is critical if we are to innovate for strategies to increase uptake and sustain use among refugee adolescents in Northern Uganda.

## 1.3 Justification

Refugees are among the most vulnerable populations in any given society. They are referred to as vulnerable because 80% of their total population is comprised of women and children (UNHCR, 2017a). Furthermore, the violence, war and discrimination which led to their displacement make them even more vulnerable. This leaves them at a high risk of facing health disparities due to a number of reasons such as their relocation and poverty.

Refugee adolescents are particularly vulnerable to difficulties that affect their sexual and reproductive health especially the girls who are left at a high risk of sexual violence, exploitation and abuse, and early or forced marriages. Several studies have shown that teenage pregnancies are higher in refugee camps than anywhere else (UNHCR, 2017). These pregnancies aggravate the already existing devastating situation in the refugee population and also have a great implication on the economy.

The Uganda Refugees Act, 2006 states that all refugee women and children shall have equal opportunities and be accorded the same treatment as the nationals (The Refugees Act, 2006), and The Uganda Adolescent Health Policy and Service Standards state that adolescents should be provided with adequate information about Adolescent Reproductive Health (ARH) services so as to promote effective use of these services including contraception (MoH, 2012). However, a large percentage of refugee adolescents continue not to use some of these ARH services like contraceptives mainly because they have limited knowledge and information about them. With a particular emphasis on contraceptive use among refugee adolescents, strategies and innovations that could help increase uptake have barely been studied, hence the limited availability of information on the strategies that could help increase contraceptives uptake. In addition, the role of peer counselling in increasing uptake of contraceptives among refugee adolescents has not been evaluated in Uganda. There is needfor us to devise innovative ways to guide self-made decisions on the methods of contraception so as to promote sustained use.

Therefore, information obtained from this study will help improve our understanding of the effect of peer counselling on contraceptive uptake and adherence among refugee adolescents in Northern Uganda. Furthermore, the study will highlight the experiences of refugee adolescents on contraception. This will help in programming and designing innovative strategies to improve contraceptive use and reduce pregnancies and their complications among refugee adolescents in Northern Uganda and similar settings.

## 1.4 Research Questions

1. What is the prevalence of modern contraceptive use among female refugee adolescents in Northern Uganda?
2. What is the effect of peer counselling on same day desire to uptake modern contraceptives among female refugee adolescents in Northern Uganda?
3. What are the rates and predictors of adherence to modern contraceptives among female refugee adolescents in Northern Uganda?
4. What are the lived experiences of female refugee adolescents on modern contraceptives in Northern Uganda?

## 1.5 General Objective

To determine the uptake of modern contraceptives among refugee adolescents in Northern Uganda, determinants, adherence, effect of peer counselling and explore the lived experiences of refugee adolescents on modern contraceptives.

## 1.6 Specific objectives

1. To determine the prevalence and factors associated with modern contraceptive use among female refugee adolescents in Northern Uganda.
2. To determine the effect of peer counselling on same day desire to uptake modern contraceptives among female refugee adolescents in Northern Uganda.
3. To determine the rates and predictors of adherence to modern contraceptives among female refugee adolescents in Northern Uganda.
4. To explore the lived experiences of female refugee adolescents on modern contraceptives in Northern Uganda.

## 1.7 Conceptual and theoretical framework

### 1.7.1 Conceptual framework

The conceptual framework describes the determinants of modern contraceptive use among refugee adolescents. The distal determinants include individual/personal factors, interpersonal factors, community and health service related factors, proximal factors include deterrents like perceived barriers and self-efficacy, motivational factors like perceived severity and susceptibility, and paths of action like perceived benefits and cues to action. The outcome of this study is uptake of modern contraceptives among refugee adolescents which in the long run will lead to reduced adolescent pregnancies. This is as shown in figure 2.

### Scope of the study

### This study will be limited to assessing individual/personal factors, inter-personal factors, community and health service related factors as well as the deterrents as the determinants of contraceptive uptake and adherence. The cues to action will also be studied under peer counselling.

**Deterrents**

**i) Perceived barriers**

**-**Misconceptions

-Inadequate knowledge

-Previous experiences

**ii) Self efficacy**

**-**Personal and vicarious experinces

**-**Social persuasion

Study IV

**Individual/personal factors**

**-**Age

-Education

-Religion

-Ethnicity

-Marital status

**Motivation/Energy**

**i) Perceived susceptibility**

-likelihood of getting pregnant

**ii) Perceived severity**

-belief that pregnancy would have serious consequences

**Interpersonal factors**

-Family

-Friends

-Partners

-Peers

**Adolescent pregnancies**

**-**Increased

-Reduced

**Community**

**-**Social and cultural norms

-Media

**Path of action**

**i) Perceived benefits**

-belief in efficacy of contraception

-belief that life is better with no pregnancy

**ii) Cues to action**

-Contraceptive counselling

-Media to increase awareness

-Reminders

Study I and III

**Health system factors**

**-**Service availability

-Quality of care

-Distance

Study II

**Fig 1.** A conceptual framework showing factors associated with contraceptive use among refugee adolescents (Adapted from (Eisen *et al*., 1985))

###

### 1.7.2 Theoretical framework

In this study, two theories of health behaviour are used to explain uptake of modern contraceptives from different perspectives. These are; the theory of reasoned action (TRA)/theory of planned behaviour (TPB) and the health belief model (HBM) as explained:

1. **Theory of reasoned action (TRA):** This theory was first developed by Martin Fishbein and Icek Ajzen in 1967 with an aim of explaining the relationship between attitude and behaviour in human actions. That is, predicting how the behaviour of individuals is based on their attitude and intentions (Rogers Gillmore *et al*., 2002). In relation to this study, this means that an adolescent’s decision to use modern contraceptives will depend on pre-existing attitude towards the modern contraceptives and their intention of using them. Adolescents with a negative attitude towards modern contraceptives are less likely to use them and vice-versa.

The theory of reasoned action also points out that attitudes and norms predict the intention of behaviour (Ajzen and Fishbein, 1980). For example, an adolescent’s attitude may lead her towards using modern contraceptives but the norms suggest otherwise, this adolescent may end up not using the contraceptives. Therefore, if we are to increase modern contraceptive among refugee adolescents, we need to strengthen a normative belief and an attitude that supports modern contraceptive use among refugee adolescents.

1. **The health belief model (HBM):** This is a psychological health behaviour change model developed in the 1950s by social psychologists to explain and predict health-related behaviours, particularly in regard to the uptake of health services (Janz and Becker, 1984). This model suggests that people's beliefs about health problems, perceived benefits of action and barriers to action, and self-efficacy explain their engagement (or lack of engagement) in those health-promoting behaviours. A stimulus, or cue to action, must also be present in order to trigger the health-promoting behaviour (Janz and Becker, 1984). This, in relation to this study means, that an adolescent will take up a modern contraceptive if she; i) feels that a negative health condition like an unwanted pregnancy can be avoided, ii) has a positive expectation that by taking the modern contraceptives she will avoid a negative condition like unwanted pregnancy and iii) believes that she can take the modern contraceptives successfully (comfortably and with confidence).

# CHAPTER TWO

# LITERATURE REVIEW

## 2.1 Modern contraceptives

Modern contraceptives are birth control or family planning methods that include the pill (oral contraceptive), injectables, implant, intra-uterine device (IUD) and condoms, both male and female. Amongst these, those commonly used by refugee adolescents are the oral contraceptive (pill) at 32.5%, followed by the male condom at 27.0%, the injectable at 21.8%, the IUD at 7.3%, the implant and the female condom at 5.3% and the emergency contraceptive at 0.6% according to a survey done in Nakivale refugee settlement in Southwest Uganda (Tanabe *et al*., 2017, UNHCR, 2011).

**Oral contraceptives (pill)**

They are available in two forms: the progestin-only pill (POP) and the combined oral contraceptive (COC) pill containing both estrogen and progestogen hormones, which are effective in preventing ovulation, implantation and hence pregnancy. They are commonly used because of their advantages like reduction in menorrhagia and dysmenorrhoea, reducing premenstrual syndrome, improving acne and endometriosis effects. They are among the most effective contraceptive with high effectiveness of over 99% in preventing pregnancy if correctly and consistently used (WHO, 2018c). They have an added advantage because they can be used even while breast feeding.

Some of the disadvantages of these pills include common side effects such as nausea and vomiting, headaches, dizziness, breast tenderness and enlargement, irregular bleeding and weight again among others. Serious side effects include severe abdominal pain, chest pain, unusual headaches, visual disturbance and leg swelling.

**Male and female condoms**

These are barrier methods of contraception that stop the sperm from meeting the egg (WHO, 2018c). If used correctly and consistently, the male condom offers 98% effectiveness while the female condom offers 90% effectiveness in preventing pregnancy (WHO, 2018c). Some of the advantages of the condoms are: you only need to use them when you have sex, they help protect both partners from Sexually Transmitted Diseases (STDs) including HIV, they are easily available and have no serious side effects. Some of the disadvantages of using condoms are that the condom may slip off or split and some people may be sensitive to the latex condoms.

**Injectable contraceptives**

There are two main types of injectable contraceptives used worldwide. These are: the combined injectable contraceptive (CIC) containing both estrogen and progestin given as a one-monthly injection, and the progestin only injectable (POI) given as a two or three monthly injection. They work by preventing ovulation, implantation and therefore prevent pregnancy.

In Uganda, the only available injectable is the Depot Medroxy Progesterone Acetate (DMPA) commonly known as Depo-Provera® and Injectaplan® given through the intramuscular or subcutaneous routes (MoH, 2014). DMPA is a highly effective contraception method with an effectiveness of 99.7% with correct and consistent use and up to 97% as commonly used (WHO, 2018c).

Some of its advantages include: convenience and ease of use, complete reversibility, reduction in menstrual cramps and the pre-menstrual syndrome, protection against endometrial cancer, and improving anaemia because of its reduction in menstrual blood flow. However, it has disadvantages like delayed return to fertility and irregular vaginal bleeding which may not be harmful (WHO, 2018c).

**Implants**

These are small, flexible rods or capsules, the size of a match stick that are placed under the skin of the upper arm that contain the hormone progestogen only. They can last either three or five years depending on the type of implant. They work by thickening the cervical mucus to block the sperm and egg from meeting and by preventing ovulation. The implants are over 99% effective in preventing pregnancy (WHO, 2018c).

The implant is commonly used because of its advantages such as: fertility returns to normal as soon as the implant is removed, it offers some protection against pelvic inflammatory disease (the mucus from the cervix may stop bacteria from entering the womb) and may offer some protection against cancer of the womb. It however has several disadvantages such as irregular vaginal bleeding, headache, acne and nausea.

**Intra-uterine devices (IUD)**

These are available in two types: the copper containing IUD and the levonorgestrel IUD. The copper IUD is a small flexible plastic device containing copper sleeves or wire that is inserted into the uterus while the levonorgestrel IUD is a T-shaped plastic device inserted into the uterus that steadily releases small amounts of levonorgestrel each day (WHO, 2018c). Both the copper and levonorgestrel IUDs are over 99% effective in preventing pregnancy.

Some of the advantages associated with using the IUDs are that the copper IUD can also be used as an emergency contraceptive, while the levonorgerstrel IUD decreases the amount of blood lost with menstruation over time. It also reduces menstrual cramps and symptoms of endometriosis. The disadvantage of the copper IUD is that it causes longer and heavier periods during the first months of use while the disadvantage of the levonorgestrel IUD is that it might cause amenorrhoea.

**Emergency contraceptive pills (ECP)**

These are pills taken to prevent pregnancy up to 5 days after unprotected sex. They are available as ulipristal acetate 30 mg or levonorgestrel 1.5 mg. The emergency contraceptive pills work by delaying ovulation and they are 99% effective (WHO, 2018c). The side effects of the ECPs include nausea and vomiting, delay in menses, irregular vaginal bleeding, abdominal pain, breast tenderness, headache, dizziness, and fatigue which usually resolve within 24 hours of pill use.

## 2.2 Modern contraceptive use among refugee adolescents

Studies have shown that modern contraceptive use among refugee adolescents is very low. A study by Amoako and Kuumuori carried out in 2016 to determine the modern contraceptive use among young women in Buduburam refugee camp in Ghana found that only 20.4% of their adolescent participants used modern contraceptives (Amoako and Kuumuori, 2016). Furthermore, a multi-country baseline study conducted by the United Nations High Commission for Refugees (UNHCR) along with the Women’s Refugee Commission (WRC) in 2011 to refocus family planning in refugee settings found the modern contraceptive prevalence rates in the selected refugee camps low i.e. 14.6% in Nakivale, Uganda; 6.8% in Eastleigh, Kenya; 5.1% in Ali Addeh, Somalia; 21.4% in Amman, Jordan; 36.9% in Cox Bazzar, Bangladesh and 34.2% in Kualar Lumpur, Malaysia (Tanabe *et al*., 2017, UNHCR, 2011). In the study by Tanabe and his colleagues in 2017, a review of the UNHCR multi-country study, it was found that participants aged 15 to 19 years from all the different refugee settings had a contraceptive prevalence rate of 4% for current use and 10.1% for previous use (Tanabe *et al*., 2017).

### 2.2.1 Factors associated with modern contraceptive use among refugee adolescents

Several factors have been reported by different researchers to affect use of modern contraceptives among refugee adolescents. These include social demographics such as age, marital status, education, occupation, partner’s level of education, parity; social cultural factors like partner’s support; service delivery factors like distance to nearest health facility.

**Age:**

Age has been reported to be associated with adolescent contraceptive use according to a study done by Nyarko in 2015. The cross sectional study by Nyarko on prevalence and correlates of contraceptive use among female adolescents in Ghana was carried out among 1037 female adolescents aged 15 to 19 years. It found that “older adolescents (between 18 and 19 years) were 3.5 times more likely to use contraceptives than the younger ones (p<0.001)” (Nyarko, 2015).

**Marital status:**

The marital status of the adolescents has been shown to be associated with uptake of contraceptives as indicated in the study by Nyarko in 2015. Nyarko reported that “married adolescents or those living with their partners were 4.8 times more likely to use contraceptives than their counterparts who were not (p<0.001)” (Nyarko, 2015).

**Education:**

The highest level of education attained has been shown to be associated with uptake of contraceptives among adolescents according to the same study by Nyarko. In that study, it was found that “adolescents who had acquired up to primary or secondary education were 7.4 and 11.5 times respectively more likely to use contraceptives than those who had not (p=0.003 and p<0.001 respectively) (Nyarko, 2015).

Another cross sectional study carried out by Amoako and Kuumuori in 2016 in Ghana to determine the prevalence of modern contraceptive use among young women in Budumburan refugee camp found that participants who had attained up to secondary education were more likely to use contraceptives than their counterparts (p=0.001) (Amoako and Kuumuori, 2016).

**Occupation/Working status:**

The occupation or working status of an adolescent has been shown to be associated with uptake of contraceptives as reported by Nyarko. Nyarko reported that “adolescents who were working were 4.8 times more likely to use contraceptives than those who were not (p<0.001)” (Nyarko, 2015).

**Partner’s level of education:**

A case control study by Lule and colleagues carried out in Kyangwali refugee settlement in Uganda to establish the determinants of contraceptive utilisation amongst teenage mothers in 2015 found that there was a statistically significant association between contraceptive use and the partner’s level of education (p=0.000, 95% CI) (Lule *et al*., 2015). Teenage mothers whose partners had attained education over primary seven were twice as likely to use contraceptives as their counterparts.

**Parity:**

The study by Lule and colleagues reported a statistically significant association between adolescent refugee contraceptive use and parity (p=0.005, 95% CI). That is, participants who had a birth order of two and below 23%were more likely to use contraceptives than their counterparts (Lule *et al*., 2015).

**Partner’s support:**

The same study by Lule and colleagues reported that contraceptive use was significantly associated with partner’s support such as escorting her to the health facility (p=0.039, 95% CI). Participants who had had support from their partners were almost twice as likely to use contraceptives as those who had had no support (Lule *et al*., 2015).

## 2.3 Adherence to modern contraceptives among refugee adolescents

Adherence is the extent to which a person's behaviour coincides with medical or health advice and in contraception, it refers to the use of a contraceptive method in an ongoing and consistent manner so as to prevent pregnancy (Jay, 1989). Therefore, for adherence to be achieved, both continuation and correct use of the contraceptives are required. Adherence can also be termed as compliance or consistency.

Adherence to modern contraceptives among refugee adolescents has not been documented, however, studies have reported adherence among non-refugee adolescents. For example; in a retrospective study conducted by Lara-Torre and Schroeder in 2002 to determine adolescent compliance and side effects with Quick Start oral contraceptive pills among 193 young females aged 22 years and below, it was found that 65% of the participants were compliant at 3 months, and only 35% compliant at 12 months (Lara-Torre and Schroeder, 2002).

A prospective cohort study by Diserens and colleagues carried out in 2017 to determine the contraceptive continuation rates among adolescents in Switzerland reported a contraceptive continuation rate of 51.9% among adolescents who had chosen the combined oestrogen-progesterone pill (Diserens *et al*., 2017).

### 2.3.1 Factors associated with adherence to modern contraceptives among refugee adolescents

Several factors have been reported to affect contraceptive adherence among adolescent. These include: age, education, parity, type of modern contraceptive used.

**Age:**

According to a retrospective study by Clare and Fraser in 2013 to determine contraceptive adherence among East Harlem adolescents, it was found that younger adolescents were at greater risk of poor adherence with contraception, and therefore used multiple methods of contraception (p=0.008) (Clare and Fraser, 2013).

**Education:**

The highest level of education attained has been shown to be associated with adherence to contraceptives among adolescents as indicated in a cross sectional study by Muhindo and colleagues to determine the predictors of contraceptive adherence among women seeking family planning at Reproductive Health Uganda, Mityana. They found that participants who had had primary or no education were 2.5 times less likely to adhere to contraceptives than their counterparts who had had secondary education (p<0.001) (Muhindo *et al*., 2015).

**Parity:**

According to a retrospective study by Lara-Torre and Schroeder in a study to determine adolescent compliance and side effects with Quick Start oral contraceptive pill, it was observed that participants who were nulliparous and nulligravid were more likely to comply or adhere to oral contraceptives compared to their counterparts (p=0.038 and 0.008 respectively) (Lara-Torre and Schroeder, 2002).

**Type of modern contraceptive used:**

The type of contraceptive used has been mentioned to be one of the factors associated with adolescent contraceptive adherence as indicated in a study by Diserens and colleagues which found that adolescents who were using the long acting reversible contraceptives such as the IUD, and the implant were more likely to adhere to the contraceptives than their counterparts who were using other contraceptive methods (p=0.011) (Diserens *et al*., 2017).

In additionally, a systematic review conducted by Usinger and others in 2016 to determine the intrauterine contraception continuation among adolescents and young women reported that, among the nine studies that met the inclusion criteria, continuation at 12 months was significantly higher among IUD users compared to participants that had used other contraceptives (p<0.001) (Usinger *et al*., 2016).

## 2.4 Effect of peer counselling on uptake of modern contraceptives

Peer counselling is a process through which trained peers provide knowledge, emotional, social and practical help to fellow peers.

According to previous research, peer counselling has shown to be effective in increasing contraceptive knowledge and hence improving contraceptive decision making among adolescents (Wilson *et al*., 2016). In a Randomised Controlled Trial (RCT) by Wilson and colleagues in 2016 evaluated the impact of peer counselling on same day desire for long acting, reversible (LARC) contraceptives among 110 female adolescents attending a family planning clinic. They found that peer counselling did not affect same day desire for LARC although peer counselled participants were more likely to report increased knowledge and positive change in attitude towards LARC;“adjusted odds ratios: 6.6 (95% confidence interval: 2.0–22.0 and 6.4 (1.6–26.8), respectively” (Wilson *et al*., 2016).

In a prospective cohort study by Jay and others in 1984 to determine the effect of peer counsellors on adolescent compliance in use of oral contraceptives among 57 females aged 14 to 19 years, it was found that at the first and second follow-ups, adolescents who had been peer counselled had a significantly lower non-compliance level than those in the nurse counselled group (p=0.038) (Jay *et al*., 1984).

## 2.5 Lived experiences of refugee adolescents on modern contraceptives

According to previous research, most of the experiences of adolescents on modern contraceptives rotate mainly around the side effects of the contraceptives and the challenges associated with the use of contraceptives. Some of the mentioned experiences are; loss of sexual desire associated with condom use, menstrual irregularities, excessive bleeding, weight gain, unintended pregnancy and stigma among others.

**Loss of sexual desire** was one of the themes that emerged as experienced with condom use according to a qualitative study by Wood and colleagues in 1997 to determine adolescent sex and contraceptive experience from the perspective of teenagers and clinic nurses in the Northern Province, South Africa. In that study, one of the adolescent participants is reported to have said, “we didn’t feel any desire when using the condom and stopped” (Wood *et al*., 1997).

**Menstrual irregularities** were the most mentioned side effect experienced with the use of injectables according to the same study by Wood et al. Many teenagers reported that they had not menstruated for weeks or months when using the injectables and “they interpreted this phenomenon in terms of blood having ‘accumulated’ or ‘clotted’, usually in the womb or abdomen but also in the head and feet, which explained why the blood would not ‘come out easily” (Wood *et al*., 1997). Some adolescents reported to have further mentioned that this state of “blood blockage” (not menstruating) was a dangerous condition that could result into illness like large or painful abdomen, swollen body, headaches, tiredness, ‘sores on the body’, weight gain and skin changes (Wood *et al*., 1997).

**Menstruating excessively** was another worry experienced by some adolescents who were using the injectables. One of the participants is reported to have said that “the excessive bleeding worried her because she felt that her blood would get finished” (Wood *et al*., 1997).

**Weight gain** was mentioned by many adolescent participants to be another issue they were experiencing while using the contraceptives. In Wood’s study, a teenager explained that her friends complained because ‘they say they have changed from a teenage weight to an adult weight and that their body is full of fluid’ (Wood *et al*., 1997).

**Unintended pregnancy** was the other issue experienced by some adolescents while on contraception as explained in Wood’s study. One of the teenagers in the study mentioned that ‘most people get pregnant while preventing, and this troubles me the most’ (Wood *et al*., 1997).

**Stigma** was also mentioned as one of the themes that arose in a qualitative study by Storehagen in 2013 to determine the adolescents’ perceptions of contraceptives and contraceptive use in Arusha, Tanzania. In that study, one of the participants is reported to have said that “unmarried adolescents using contraceptives were stigmatised” and as a consequence, they had challenges with accessing contraceptives at the health centres (Storehagen, 2013).

# CHAPTER THREE

# METHODS

## 3.1 Study setting

This study will be carried out from Palabek refugee settlement located in the Northern region of Uganda in Lamwo district. Palabek is the newest refugee settlement established in Uganda in April 2017 and is currently hosting more than 32,000 South Sudanese refugees (UNHCR, 2018), 86% of whom are women and children (UNHCR, 2017b).

Palabek refugee settlement has four health centres all of which provide family planning services freely to all refugees and to the host community. Some of the family planning services provided include: contraceptive counselling, giving out of oral contraceptives and condoms, insertion and removal of intra-uterine devices (IUDs) and implants, and giving of injectable contraceptives.


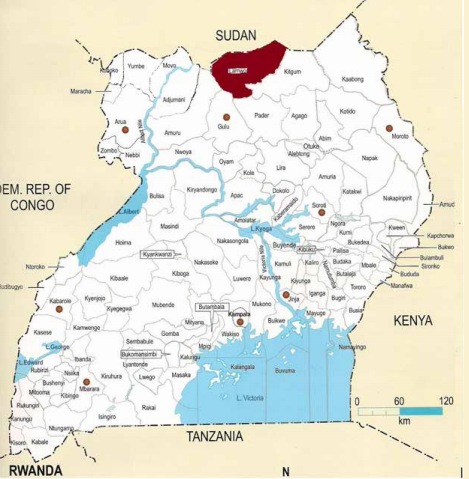

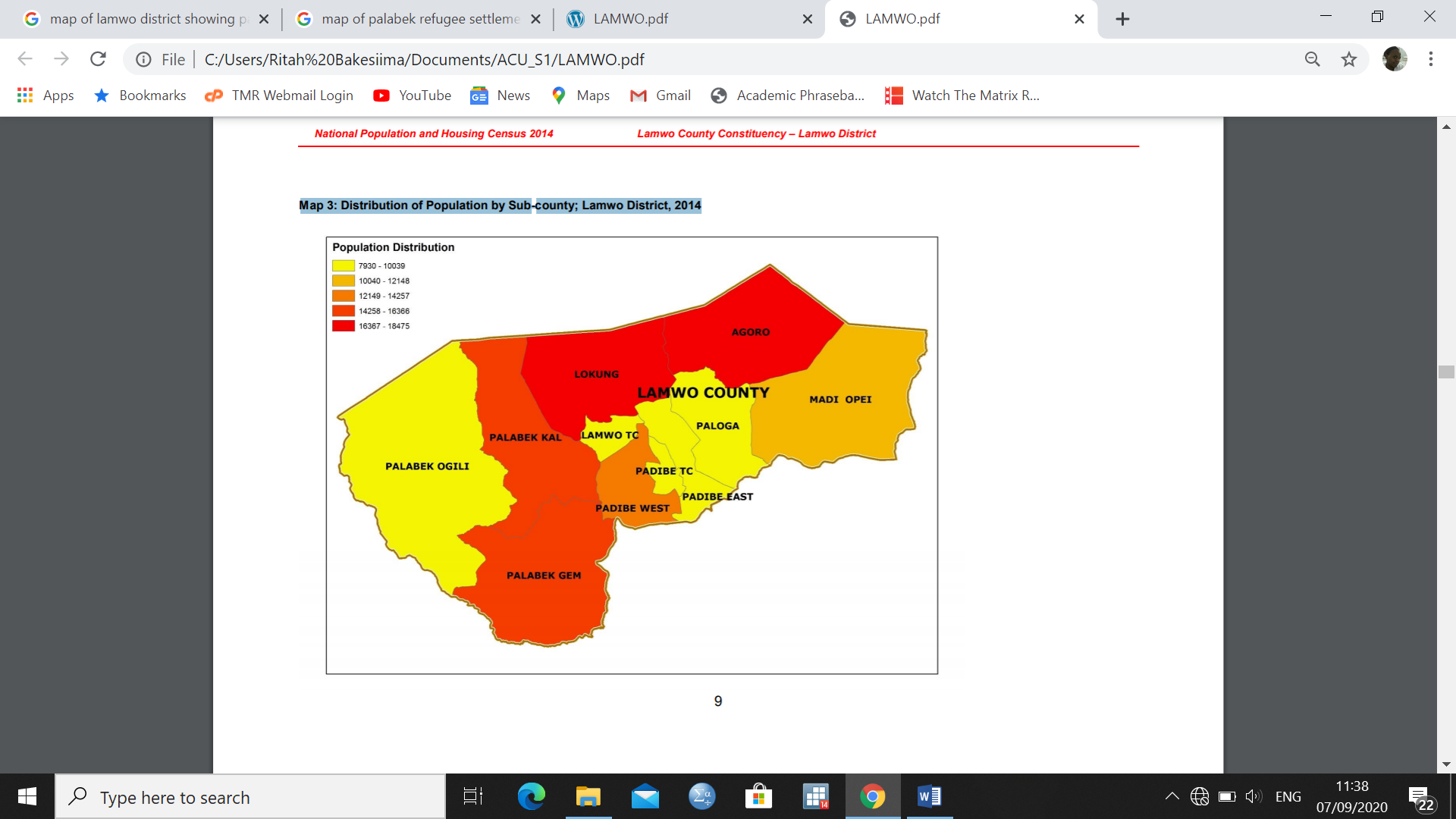


**Fig 2.** Map of Uganda showing the study site

Base maps retrieved from: (UNDP, 2019) <https://www.ugandainvest.go.ug/wp-content/uploads/2019/06/UNDPUg1720DistrictProfile_Lamwo.pdf>

Source of map data: (UBOS, 2014) <https://www.ubos.org/wp-content/uploads/publications/2014CensusProfiles/LAMWO.pdf>

## 3.2 Study design

This study will have a mixed-method study design including both qualitative and quantitative methods. It will be carried out in four sub-studies as shown in the table 1.

**Table 1:** Summary of methods

| **Objective** | **Study design** | **Study population** | **Methods** |
| --- | --- | --- | --- |
| To determine the prevalence and factors associated with modern contraceptive use among refugee adolescents in Northern Uganda | Cross sectional | Female refugee adolescents aged 10 - 19 years who are sexually active or in-union in Palabek refugee settlement between May and July 2019 | Structured questionnaire |
| To determine the effect of peer counselling on uptake of modern contraceptives among refugee adolescents in Northern Uganda | Randomised controlled trial | Female refugee adolescents aged 15-19 years who are sexually active or in-union in Palabek refugee settlement between May and July 2019 | Intervention: peer counselling  Control: Routine counselling  Structured questionnaires |
| To determine the rates and predictors of adherence to modern contraceptives among refugee adolescents in Northern Uganda | Prospective single cohort | Female refugee adolescents aged 15-19 years in Palabek refugee settlement between May and December 2019 who are new acceptors of contraceptives | Structured questionnaires  Follow up at 1, 3, and 6 months |
| To explore the lived experiences of refugee adolescents on modern contraception in Northern Uganda | Phenomenological study | Female refugee adolescents aged 15-19 years in Palabek refugee settlement who will have used contraceptives for at least 6 months in February 2019 | In-depth interviews |

Participant recruitment

Assessed for eligibility

Randomisation

Contraceptive non-users

**Study I**

Eligible and scheduled for enrolment

Peer counselling

Routine counselling

New acceptors

1, 3, and 6 months follow up

6 month adherers

Study IV

**Study IV**

**Study II**

**Study III**

Ineligible

Contraceptive users

Non-acceptors Non-acceptors

Non-adherent

**Fig 3.** Participant flowchart

## 3.3 Sub study 1

3.3.1 Objective: To determine the prevalence and factors associated with modern contraceptive use among female refugee adolescents in Northern Uganda.

3.3.2 Study design: This will be a cross sectional study carried out in Palabek refugee settlement between May and July 2019.

3.3.3 Population:

**Target population:**

All female refugee adolescents aged 10 to 19 years who are sexually active or in-union in Northern Uganda.

**Accessible population:**

All female refugee adolescents aged 10 to 19 years who are sexually active or in-union in Palabek refugee settlement in Lamwo district.

**Study population:**

All female refugee adolescents aged 10 to 19 years who are sexually active or in-union in the Palabek refugee settlement between May and July 2019 who will consent to participate in the study.

### 3.3.4 Eligibility criteria:

**Inclusion criteria:**

Refugee adolescents will be included in this study if they are:

1. Females aged 10 to 19 years,
2. Sexually active or in-union,
3. Settled within Palabek refugee settlement between May and July 2019 and consent to participate in the study.

**Exclusion criteria:**

Refugee adolescents will be excluded from this study if they:

1. Cannot comprehend either English, Nuer or Arabic
2. Are physically or mentally unable to adhere to study procedures.

3.3.5 Sample size: To determine the sample size for the prevalence of modern contraceptive use, the Kish Leslie formula is used as below:

**** (Kish, 1965)

For:

n is the sample size

Z is the standard normal value corresponding to the level of significance (1.96 at 95% level of significance)

p – Expected prevalence in population based on previous studies or pilot studies (assuming a prevalence of 50%)

d – Absolute error or precision (0.05)

Based on the above formula, the estimated sample size is 385 female adolescents, and when the 10% non response is factored in, the minimum sample size to answer this objective will be 424 female adolescents.

To determine the factors associated with modern contraceptive use, the formula below is used:

Where;

Z_α/2_ is the standard normal value corresponding to the level of significance (e.g. for a confidence level of 95%, α is 0.05 and the critical value is 1.96),

Z_β_ is the standard normal value corresponding to the power of the study (e.g. for a power of 80%, β is 0.2 and the critical value is 0.84),

p_1_ is the proportion of adolescents who are currently married and are using modern contraceptives (20.7% - (UDHS, 2016)) while p_2_ is the proportion of unmarried sexually active adolescents using modern contraceptives (40.3% - (UDHS, 2016)).

q_1_ is the proportion of adolescents in Uganda who are currently married (5.6% - (UDHS, 2016).

q_2_ is the proportion of unmarried sexually active adolescents in Uganda (94.4% - (UDHS, 2016)).

Based on the above formula, the calculated sample size is 839 participants. Since the sample size for determining the factors associated with modern contraceptive use is bigger, it will be taken as the overall sample size for this sub-study. Therefore, 839 female adolescents will be required to answer this objective.

3.3.6 Sampling procedure: Adolescents who are eligible and consent to participate in the study will be consecutively enrolled into the study with the help of camp commanders and zone leaders who will be our contact persons. The zone leaders will help approach adolescents in the camp and request them to participate in the study. Those who will accept to participate will be screened for eligibility and the study protocol explained to each of the eligible respondents. Thereafter, written informed consent will be obtained from them and questionnaires administered by the interviewers.

3.3.7 Data collection: Data will be collected using an interviewer administered questionnaire. The questionnaires will be used to obtain information on social demographics, sexual and reproductive history, spousal information and knowledge and use of modern contraceptives. Two trained research assistants will conduct the interviews.

**Study variables**

***Independent variables:***

1. Social demographics include age, highest level of education attained, religion, ethnicity, marital status, occupation, years in the camp.
2. Sexual and reproductive history such as age at first sex, number of sexual partners, parity, current and desired number of children
3. Knowledge of contraceptives
4. Contraceptive use/non-use and reasons for non-use.

***Dependent variables:***

1. Primary outcome variable: Contraceptive use (prevalence of female adolescents currently using any modern contraceptive method).
2. Secondary outcome variables: Types of modern contraceptives used, duration of use of modern contraceptives, source of modern contraceptive.

**Data collection activities:**

1. Initial visits to the study sites will be done by the principal investigator to get the necessary approvals and meet with the study team.
2. Study materials and tools will be purchased and prepared.
3. Research assistant recruitment, training, familiarization and review of study tools will also be done.
4. Pre-testing of the questionnaires, practising interviews and the recruitment process will also be done.
5. The principal investigator along with two research assistants will then collect data at scheduled times for a period of two months.

### 3.3.8 Data analysis

Data will be analysed using STATA version 13.0. All continuous variables will be summarised as means and standard deviations if they are normally distributed, and as medians and ranges if skewed. Categorical variables will be summarised as percentages and proportions. Modern contraceptive use will be analysed as a categorical variable, with use of modern contraceptives coded as “1” and non-use as “0”.

***Prevalence*** of modern contraceptive use among refugee adolescents will be calculated as the percentage of refugee adolescents currently using modern contraceptives over the total number of participants in the study.

***Factors associated with modern contraceptive use*** will be assessed using the logistic regression model. Bivariate analysis will be done by fitting a model for all the independent variables with the outcome, modern contraceptive use. All the variables that give a p-value ≤0.2 at the bivariate analysis will be considered for multivariate analysis.

At the multivariate analysis, the logistic regression model will be run for all the variables which meet criteria for it, and those which will be found to be significant at this stage (p<0.05) will be used to form two-way product terms. These product terms will be used in the assessment of interaction using the chunk test and each interaction term will be individually checked for significance using the test parameter approach by dropping the interaction terms one at a time starting with the least significant while the remaining ones will be checked if they have attained significance. Where necessary, confounding will be assessed for and a variable will be considered as a confounder if it causes a greater than or equal to 10% change in the prevalence ratio of modern contraceptive use. Odds ratios along with their 95% confidence intervals will be reported, and statistical significance reported at p<0.05.

**Publication:** 1 manuscript planned.

## 3.4 Sub-study II:

3.4.1 Objective: To determine the effect of peer counselling on same day desire to uptake of modern contraceptives among female refugee adolescents in Northern Uganda.

3.4.2 Study design: This will be a randomised, controlled, outcome assessor blinded, superiority trial with two-parallel groups in a 1:1 allocation ratio carried out in Palabek refugee settlement between May and July 2019.

### 3.4.3 Hypothesis:

Alternative: Individual peer counselling of refugee adolescents in Northern Uganda will result into a 30% difference in uptake of modern contraceptives compared to routine counselling.

Null: There is no difference in contraceptive uptake among refugee adolescents who receive individual peer counselling compared to those who receive routine counselling.

### 3.4.4 Study population:

**Target population:**

All female refugee adolescents aged 15 to 19 years who are sexually active or in-union in Northern Uganda.

**Accessible population:**

All female refugee adolescents aged 15 to 19 years who are sexually active or in-union in Palabek refugee settlement in Lamwo district.

**Study population:**

All female refugee adolescents aged 15 to 19 years who are sexually active or in-union in Palabek refugee settlement and will not be using modern contraceptives between May and July 2019 and consent to participate in the study.

### 3.4.5 Eligibility criteria:

**Inclusion criteria:**

Refugee adolescents will be included in this study if they are:

1. Females aged 15 to 19 years,
2. Sexually active or in-union,
3. Settled within Palabek refugee settlement between May and July 2019,
4. Currently not using any modern contraceptives and consent to participate in the study.

**Exclusion criteria:**

Refugee adolescents will be excluded from this study if they:

1. Cannot comprehend either English, Nuer or Arabic
2. Are physically or mentally unable to adhere to study procedures.

3.4.6 Sample size: The formula below is used to determine the sample size for an RCT as shown below:

n = [(Z_α/2_ + Z_β_)^2^ × ((p1 (1-p1) + (p2 (1-p2)))] (Hayes and Bennett, 1999)

(p1 - p2)^2^

For:

n = sample size required in each group,

Z_α/2_ is the standard normal value corresponding to the level of significance (e.g. for a confidence level of 95%, α is 0.05 and the critical value is 1.96),

Z_β_ is the standard normal value corresponding to the power of the study (e.g. for a power of 80%, β is 0.2 and the critical value is 0.84),

p1 = proportion of contraceptive uptake among adolescents on standard counselling (0.204 - (Amoako and Kuumuori, 2016)).

p2 = proportion of contraceptive uptake among adolescents on peer counselling (0.304 assuming a 10% difference between the two proportions)

p1 - p2 = clinically significant difference = 0.10

Basing on the above formula, the sample size required for each group will be 294, and therefore the total sample size for this sub-study will be 588 female adolescents.

Therefore, a sample size of 588 female adolescents, 294 in each arm, is sufficient to detect a clinically important difference of 10% between groups in improving uptake of contraceptives using a two-tailed z-test of proportions between two groups with 80% power and a 5% level of significance.

3.4.7 Sampling procedure: All adolescents that are eligible and consent to participate in the study will be randomly assigned to either the intervention or the control group. The camp commanders and zone leaders will be our contacts and they will help approach adolescents in the different zones and request them to participate in the study. Those who will accept to participate will be screened for eligibility and the study protocol explained to each of the eligible respondents. Thereafter, written informed consent will be obtained from them and questionnaires administered by the interviewers.

3.4.8 Intervention: Peer counselling will involve the use of trained peers to counsel fellow adolescents on the different contraception methods with the aid of a standardised contraceptive counselling tool which will be administered to all participants. Peer counsellors will also explain to the adolescents all the information there is to know about contraceptives for example method advantages and disadvantages, effectiveness, and also dispel the misconceptions and myths that the adolescents could be having about contraceptives. Physical models of the different contraceptives will also be shown to the participants during counselling and descriptions of the insertion demonstrated where necessary.

***Peer counsellors*:** Five female adolescent will be selected from the study site to serve as peer counsellors based on their verbal interaction abilities, leadership skills, and their social and sexual maturity, preferably between 17 and 19 years of age. Peer counsellors will also be given organised training sessions in conversational and interaction skills, observational skills, decision making, formal counselling, confidentiality, problem solving and birth control.

3.4.9 Control: The control for this study will be routine contraceptive counselling which is given at the health facilities within the refugee camp as the standard of care. This counselling is usually offered by the nurses at these health centres as a single session involving all women who are seeking contraceptives that day. This counselling is not guided with a counselling tool, rather it is done off the cuff by the nurses.

3.4.10 Randomisation: Participants will be randomly assigned to either the control or intervention group with a 1:1 ratio using permuted blocks of random sizes. The block sizes will not be disclosed to ensure allocation concealment. The person generating the randomisation sequence will not participate in the execution of the randomisation.

**Allocation concealment:** This will be achieved by enclosing assignments in sequentially numbered opaque sealed envelopes to ensure that the person randomising does not know what the next treatment allocation is. In addition, central randomisation (using a randomisation point remote from the trials location) will be used.

**Blinding:** Assessment of uptake of modern contraceptives among the participating refugee adolescents will be conducted by an assessor blinded to the allocation arm of the participant. Due to the nature of the intervention, neither the participants nor the investigators can be blinded. However, they will be strongly inculcated not to disclose the allocation status to the outcome assessors. An external person will be used to enter the data so that the data analysts may not be aware of the allocation.

3.4.11 Data collection: Participants who will be assigned to the intervention arm will receive peer counselling by trained peers while those assigned to the control arm will receive the routine counselling provided at the health centres. Data will be collected using an interviewer administered questionnaire after receiving the counselling. The questionnaires will be used to obtain information on social demographics, sexual and reproductive history, and knowledge and use of modern contraceptives. Two trained research assistants will conduct the interviews.

**Study variables**

***Predictor variables:***

1. Social demographics include age, highest level of education attained, religion, ethnicity, marital status, occupation, years in the camp.
2. Sexual and reproductive history like age at first sex, number of sexual partners, parity.
3. Knowledge of contraceptives

***Outcome variables:***

1. Primary outcome variable: Desire to uptake modern contraceptives
2. Secondary outcome variables: Type of modern contraceptive desired

### 3.4.12 Data analysis

STATA version 13.0 will be used for analysis, where continuous variables will be summarised as means and standard deviations if they are normally distributed, and as medians and ranges if skewed, while categorical variables will be summarised as percentages and proportions.

***Uptake of modern contraceptives*** will be analysed as a categorical variable, with willingness to take up contraception coded as “1” and unwillingness “0”. Percentages of acceptors in the two groups will be obtained and the Chi-square test used to compare contraceptive uptake between the two groups.

***Factors influencing contraceptive uptake*** will be estimated using the logistic regression model. Bivariate analysis will be done by fitting a model for all the independent variables with the outcome. All the variables that give a p-value ≤0.2 at the bivariate analysis will be considered for multivariate analysis.

At the multivariate analysis, the logistic regression model will be run for all the variables which meet criteria for it, and those which will be found to be significant at this stage (p<0.05) will be used to form two-way product terms. These product terms will be used in the assessment of interaction using the chunk test and each interaction term will be individually checked for significance using the test parameter approach by dropping the interaction terms one at a time starting with the least significant while the remaining ones will be checked if they have attained significance. Where necessary, confounding will be assessed for and a variable will be considered as a confounder if it causes a greater than or equal to 10% change in the prevalence ratio of modern contraceptive use. Odds ratios along with their 95% confidence intervals will be reported, and statistical significance reported at p<0.05.

**Publication:** 1 manuscript planned

## 3.5 Sub-study III:

3.5.1 Objective: To determine the rates and predictors of adherence to modern contraceptives among refugee adolescents in Northern Uganda.

3.5.2 Study design: This will be a prospective single cohort study carried out in Palabek refugee settlement between January and August 2019.

### 3.5.3 Population:

**Target population:**

All female refugee adolescents aged 15 to 19 years in Northern Uganda.

**Accessible population:**

All female refugee adolescents aged 15 to 19 years in Palabek refugee settlement in Lamwo district.

**Study population:**

All female refugee adolescents aged 15 to 19 years in Palabek refugee settlement between May and December 2019 who are new acceptors of modern contraceptives and consent to participate in the study.

### 3.5.4 Eligibility criteria:

**Inclusion criteria:**

Refugee adolescents will be included in this study if they are:

1. Females aged 15 to 19 years,
2. Settled within Palabek refugee settlement between May and December 2019,
3. New acceptors of modern contraceptives and consent to participate in the study.

**Exclusion criteria:**

Refugee adolescents will be excluded from this study if they:

1. Cannot comprehend either English, Nuer or Arabic
2. Are physically or mentally unable to adhere to study procedures.

3.5.5 Sample size: To determine the sample size for the rate of adherence to contraception, the Kish Leslie formula will be used as below:

(Kish, 1965)

For:

N is the sample size

Z is the standard normal value corresponding to the level of significance (1.96 at 95% level of significance)

p – Expected prevalence in population based on previous studies or pilot studies (35% - (Lara-Torre and Schroeder, 2002))

d - Absolute error or precision (0.05)

Based on the above formula, the estimated sample size is 350 female adolescents, and when the 10% loss to follow up is factored in, the minimum sample size to answer this objective will be 385 female adolescents.

To determine the sample size for the factors associated with adherence to contraception, the formula below was used:

Where;

Z_α/2_ is the standard normal value corresponding to the level of significance (e.g. for a confidence level of 95%, α is 0.05 and the critical value is 1.96),

Z_β_ is the standard normal value corresponding to the power of the study (e.g. for a power of 80%, β is 0.2 and the critical value is 0.84),

p_1_ is the proportion of adolescents who discontinued on a long acting reversible contraceptive (0.35 - (Sznajder *et al*., 2017) while p_2_ is the proportion of adolescents who discontinued on other contraceptives (0.60 - assuming a 25% difference between the two groups).

q_1_ is the proportion of adolescents in Uganda on a long acting reversible contraceptive (0.126 - (Tanabe *et al*., 2017)).

q_2_ is the proportion of adolescents in Uganda on non-long acting reversible contraceptives (0.874 - (Tanabe *et al*., 2017)).

Based on the above formula, the calculated sample size is 274 female adolescents. Since the sample size for the prevalence study is bigger, it will be taken as the overall sample size for this sub study. Therefore, 385 female adolescents will be required for this sub-study.

3.5.6 Sampling procedure**:** All adolescents that are eligible and consent to participate in the study will be consecutively enrolled into this study. The camp commanders and zone leaders will be our contacts and they will help approach adolescents in the community and request them to participate in the study. Those who will accept to participate will be screened for eligibility and the study protocol explained to the each of the eligible respondents. Thereafter, written informed consent will be obtained from them and questionnaires administered with the help of an interviewer.

3.5.7 Data collection: Data will be collected using an interviewer administered questionnaire to obtain information on social demographics, sexual and reproductive history, and type of contraceptive used. Information will be collected at baseline, 1, 3, and 6 months with follow-up questionnaires administered by an interviewer at each follow up. Two trained research assistants will conduct the interviews.

**Study variables**

***Predictor variables:***

1. Social demographics include age, highest level of education attained, religion, ethnicity, marital status, occupation, years in the camp.
2. Sexual and reproductive history like age at first sex, number of sexual partners, parity.
3. Knowledge of contraceptives.

***Outcome variables:*** Adherence to modern contraceptives.

3.5.8 Follow up strategy: A contact locator will be developed which will contain information like the address and telephone numbers of respondents and their ‘significant other’ who could be a relative, spouse or friend, and head of household identification. These will be recorded to enhance contact and follow up of respondents. In addition, each respondent shall be given a unique identification number and a small card with their particulars which will be presented at the follow-up visit. In the event of a missed appointment, they will be contacted by phone or home visited within 2 weeks of the missed appointment. A respondent will be considered as lost to follow up if we fail to trace her for 3 months.

3.5.9 Non-adherence scale: Non-adherence will be measured with a three-factor Gutmann scale that consists of the following; i) whether the subject became pregnant during the previous month, ii) whether she missed her appointment and iii) whether she missed taking three or more oral contraceptives during the month.

3.5.10 Data analysis: Data will be analysed using STATA version 13.0 where continuous variables will be summarised as means and standard deviations if they are normally distributed, and as medians and ranges if skewed, while categorical variables will be summarised as percentages and proportions.

***Adherence to contraceptives*** will be analysed as a categorical variable, with adherence coded as “1” and non-adherence as “0”. Percent adherence at the different follow up times will then be obtained.

***Factors associated with adherence*** will be estimated using the logistic regression model. Bivariate analysis will be done by fitting a model for all the independent variables with the outcome. All the variables that give a p-value ≤0.2 at the bivariate analysis will be considered for multivariate analysis.

At the multivariate analysis, the logistic regression model will be run for all the variables which meet criteria for it, and those which will be found to be significant at this stage (p<0.05) will be used to form two-way product terms. These product terms will be used in the assessment of interaction using the chunk test and each interaction term will be individually checked for significance using the test parameter approach by dropping the interaction terms one at a time starting with the least significant while the remaining ones will be checked if they have attained significance. Where necessary, confounding will be assessed for and a variable will be considered as a confounder if it causes a greater than or equal to 10% change in the prevalence ratio of modern contraceptive use. Odds ratios along with their 95% confidence intervals will be reported, and statistical significance reported at p<0.05.

**Publication:** 1 manuscript planned.

## 3.6 Sub-study IV:

3.6.1 Objective: To explore the lived experiences of female refugee adolescents on modern contraceptives in Northern Uganda.

3.6.2 Study design: This will be a phenomenological study carried out in Palabek refugee settlement in February 2020.

### 3.6.3 Population:

**Target population:**

All female refugee adolescents aged 10 to 19 years in Northern Uganda.

**Accessible population:**

All female refugee adolescents aged 10 to 19 years in Palabek refugee settlement in Lamwo district.

**Study population:** Female refugee adolescents aged 10 to 19 years in Palabek refugee settlement who will have used modern contraceptives for at least 6 months in February 2020 and consent to participate in the study.

**Sampling procedure:** Participants will be purposively selected into the study basing on participants’ characteristics like age (younger – 15 to 17 years, and the older adolescents – 18 and 18 years), marital status (married and unmarried), highest level of education attained (educated and no formal education), contraceptive type (long-acting reversible and short acting), duration of use of contraceptives (short term and long term) and on adherence (continued and discontinued on modern contraceptives). Participants will be approached using face to face approaches and asked to participate in the study.

3.6.4 Sample size: The required sample size will be obtained basing on data saturation, a point at which no new information is being obtained from the participants (Corbin and Strauss, 2014) during the individual in-depth interviews.

3.6.5 Data collection: Data will be collected using in-depth interviews from within the communities. These interviews will be conducted by qualitative interview experts. Interview questions, prompts and guides will be provided by the Principal Investigator, and these will be pretested before use. Audio recording will be done for all the interviews and later transcribed by a transcription expert who is well conversant with the language used. Field notes will also be taken by the interviewer during the interview process.

3.6.6 Data analysis**:** Collected data will be entered in OPENCODE computer software for proper management. This software will be used in sorting and coding data, retrieval, systematic searching, and storage of data. Data analysis will be done using thematic analysis. This will be done by identifying key issues, which will be indexed into textual data, followed by constant comparison of the coded data. Themes will be inductively obtained from the data. Analytical categories will then be formed to describe and explain the patterns, and finally the data can be communicated and explained using diagrams and illustrative quotes.

**Publication:** 1 manuscript planned.

## 3.7 Data management

**Data storage:** Data will be collected and recorded at the point of contact on the study data collection forms. All data will be checked for accuracy, completeness and consistency at the close of each day, and any identified errors will be corrected immediately. The data will then be double entered and backed up on another external device and in space which will all be password protected and only accessible to authorised personnel. All subsequent analysis will be done on a copy of the frozen data using STATA version 13.0. All study related source documents for each patient will be placed into a study file and kept in a secure file cabinet in the study rooms.

**Data quality control:**

***Training:*** All research assistants will be trained on all study procedures beforehand by the Principal Investigator.

***Reproducibility:*** Questionnaires will be translated to Arabic and Dinka which are the widely spoken languages in the refugee camp and back translated using qualified and competent persons to ensure that the meaning is not altered. These questionnaires will also be pretested using 10 women from the study site to check for its feasibility as a data collection tool, and adjustments made where necessary.

***Manuals and SOPs:*** Manuals of Standard Operating Procedures will be developed for all study related procedures and protocols. The Principal Investigator and the research assistants will be trained in the SOPs and study protocol. Regular meetings and retraining will occur throughout the study in order to maintain standards of data collection.

Questionnaires will be checked for completeness daily before leaving the field, and will thereafter be double entered, cleaned and coded. All data will be kept in both hard and soft copy, and all soft copy data will be backed up on other computers. Data will then be transferred to STATA version 13.0 for analysis

## 3.8 Ethical considerations

Ethical approval will be obtained from the Makerere University School of Medicine Higher Degrees Research and Ethics Committee. Administrative clearance will also be sought from the Department of Refugees, Office of the Prime minister. Written informed consent will be sought from all the participants before enrolment into the study and additional assent obtained from those below 18 years of age. Confidentiality will be maintained by using number codes instead of the participant’s names.

#

# REFERENCES

AJZEN, I. & FISHBEIN, M. 1980. Understanding attitudes and predicting social behavior. *Englewood Cliffs, NJ: Prentice-Hall*.

AMOAKO, D. & KUUMUORI, J. 2016. Modern contraceptive use among young women in Buduburam refugee camp in Ghana.

AZEVEDO, W. F. D., DINIZ, M. B., FONSECA, E. S. V. B. D., AZEVEDO, L. M. R. D. & EVANGELISTA, C. B. 2015. Complications in adolescent pregnancy: systematic review of the literature. *Einstein (São Paulo),* 13**,** 618-626.

BLANC, A., CURTIS, S. & CROFT, T. 2002. Monitoring contraceptive continuation: links to fertility outcomes and quality of care, Studies in Family Planning. 33**,** 127-140.

BLANC, A., TSUI, A., CROFT, T. & TREVITT, J. 2009. Patterns and trends in adolescents' contraceptive use and discontinuation in developing countries and comparisons with adult women. *35,* 2**,** 63-71.

BOAMAH, E., ASANTE, K. & MAHAMA, E., MANU G, AYIPAH E, ADENIJI E, OWUSU-AGYEI S 2014. Use of contraceptives among adolescents in Kintampo, Ghana: a cross-sectional study. *OAJC,* 5**,** 7-15.

CLARE, C. & FRASER, C. 2013. Contraception Adherence among East Harlem Adolescents. *Gynecol Obstet,* 3.

CORBIN, J. & STRAUSS, A. 2014. Basics of qualitative research: Techniques and procedures for developing grounded theory. *Sage publications*.

CROWNE, S., GONSALVES, K., BURRELL, L., MCFARLANE, E., AND & DUGGAN, A. 2012. Relationship between birth spacing, child maltreatment, and child behavior and development outcomes among at-risk families. *Maternal andChild Health Journal,* 16**,** 1413-1420.

DEHLENDORF, C., KRAJEWSKI, C., & & BORRERO, S. 2014. Contraceptive Counseling: Best Practices to Ensure Quality Communication and Enable Effective Contraceptive Use. . *Clinical Obstetrics and Gynecology,* 57**,** 659-673.

DISERENS, C., QUACH, A., MATHEVET, P., BALLABENI, P. & JACOT-GUILLARMOD, M. 2017. Adolescents' contraception continuation in Switzerland: a prospective observational study. *Swiss Med Wkly,* 147**,** w14504.

EISEN, M., ZELLMAN, G., , & MCALISTER, A. 1985. A Health Belief Model Approach to Adolescents’ Fertility Control: Some Pilot Program Findings. *Health Education Quarterly,* 12**,** 185-210.

FP2020 2015. Contraceptive discontinuation: reasons, callenges and solutions.

GIVEN, L. 2008. "Lived Experience". The SAGE Encyclopedia of Qualitative Research Methods. SAGE Publications. 1 and 2.

HAYES, R. J. & BENNETT, S. 1999. Simple sample size calculation for cluster-randomized trials. *Int J Epidemiol,* 28**,** 319-26.

JANZ, N. K. & BECKER, M. H. 1984. The Health Belief Model: A Decade Later. *Health Education & Behavior,* 11**,** 1-47.

JAY, M., DURANT, H. R., SHOFFITT, T., CHARLES, W. L. W. C. & AND LIII, F. I. 1984. Effect of peer counsellors on adolescent compliance in use of oral contraceptives. *Pediatrics,* 73.

JAY, M. S., R.H. DURANT, AND I.F. LITT 1989. Female adolescents' compliance with contraceptive regimens. *Pediatr Clin North Am,* 36**,** 731-746.

KABAGENYI, A., HABAASA, G. & RUTAREMWA, G. 2016. Low Contraceptive Use among Young Females in Uganda: Does Birth History and Age at Birth have an Influence? Analysis of 2011 Demographic and Health Survey. *Journal of contraceptive studies,* 1**,** 4.

KAUNITZ, A. 2017. Contraceptive coumselling and selection.

KISH, L. 1965. Survey Sampling. New York: John Wiley and Sons, Inc. 78-94.

LARA-TORRE, E. & SCHROEDER, B. 2002. Adolescent compliance and side effects with Quick Start initiation of oral contraceptive pills. *Contraception,* 66**,** 81-5.

LEMANI, C., TANG, J., KOPP, D., PHIRI, B., KUMVULA, C. & CHIKOSI, L., ET AL. 2017. Contraceptive uptake after training community health workers in couples counseling: A cluster randomized trial. *PLoS ONE* 12.

LULE, H., ECHORU, I., NNABAGULANYI, M. & MULUMBA, R. 2015. *Determinants of contraceptive utilisation amongst teenage mothers: a case control study in kyangwali refugee settlement (Uganda)*.

MOH 2012. Adolescent health policy guidelines and service standards.

MOH 2014. Family planning 2020 (<http://www.familyplanning2020.org/entities/80)>.

MUHINDO, R., NANKUMBI, O. J., SARA GROVES. & MICHELENE, C. 2015. Predictors of Contraceptive Adherence among Women Seeking Family Planning Services at Reproductive Health Uganda, Mityana Branch. *International Journal of Population Research,* 10.

NYARKO, S. 2015. Prevalence and correlates of contraceptive use among female adolescents in Ghana. *BMC Women's Health* 16.

OKANLAWON, K., REEVES, M. & AGBAJE, O. F. 2010. Contraceptive Use: Knowledge, Perceptions and Attitudes of Refugee Youths in Oru Refugee Camp, Nigeria. *African Journal of Reproductive Health / La Revue Africaine de la Sant&#xe9; Reproductive,* 14**,** 16-25.

ROGERS GILLMORE, M., ARCHIBALD, M., MORRISON, D., WILSDON, A., WELLS, E., HOPPE, M., NAHOM, D. & MUROWCHICK, E. 2002. Teen Sexual Behavior: Applicability of the Theory of Reasoned Action. *Journal of Marriage and Family,* 64**,** 885-897.

RUTSTEIN, S. 2005. Effects of preceding birth intervals on neonatal, infant and under-five years mortality and nutritional status in developing countries: evidence from the Demographic and Health Surveys. *International Journal of Gynecology & Obstetrics,* 89**,** S7-S24.

SEDGH, S., SINGH, S. & HUSSEIN, R. 2014. Intended and unintended pregnancies worldwide in 2012 and recent trends, Studies in Family Planning. 45**,** 301-314.

STOREHAGEN, L. 2013. From adolescents own perspectives: Perceptions of contraceptives and contraceptive use : A qualitative study in Arusha, Tanzania.

SZNAJDER, K. K., TOMASZEWSKI, K. S., BURKE, A. E. & TRENT, M. 2017. Incidence of Discontinuation of Long-Acting Reversible Contraception among Adolescent and Young Adult Women Served by an Urban Primary Care Clinic. *Journal of Pediatric and Adolescent Gynecology,* 30**,** 53-57.

TANABE, M., MYERS, A., BHANDARI, P., CORNIER, N., DORAISWAMY, S. & KRAUSE, S. 2017. Family planning in refugee settings: findings and actions from a multi-country study. *Conflict and Health,* 11**,** 9.

THE REFUGEES ACT 2006. Uganda: The Refugee Act 2006 [Uganda], Act 21

UDHS 2016. Demographic and Health Survey 2016, Key Indicators Report.

UNFPA 2017a. Adolescent pregnancy.

UNFPA 2017b. From family planning to "future planning".

UNHCR 2011. Baseline Study: Documenting Knowledge, Attitudes and Practices of Refugees and the Status of Family Planning Services in UNHCR’s Operations in Nakivale Refugee Settlement, Uganda.

UNHCR 2016. Pregnant and on the run.

UNHCR 2017a. Comprehensive refugee response framework Uganda, The way forward.

UNHCR 2017b. UNHCR Uganda Factsheet - June 2017.

UNHCR 2018. Uganda Refugee Response Monitoring Settlement Fact Sheet: Palabek / January 2018.

USINGER, K. M., GOLA, S. B., WEIS, M. & SMALDONE, A. 2016. Intrauterine Contraception Continuation in Adolescents and Young Women: A Systematic Review. *Journal of Pediatric and Adolescent Gynecology,* 29**,** 659-667.

WHO 2007. Contraceptive counselling and compliance.

WHO 2014. Adolescent pregnancy.

WHO 2017. Improving family planning service delivery in humanitarian crises.

WHO 2018a. Adolescent health and developmemt.

WHO 2018b. Adolescent pregnancy.

WHO 2018c. Family planning/Contraception.

WILSON, S. F., DEGAIFFIER, N., RATCLIFFE, S. J. & SCHREIBER, C. A. 2016. Peer counselling for the promotion of long-acting, reversible contraception among teens: a randomised, controlled trial. *Eur J Contracept Reprod Health Care,* 21**,** 380-7.

WOOD, K., MAEPA, J. & JEWKES, R. 1997. Adolescent sex and contraceptive experiences: perspectives of teenagers and clinic nurses in the Northern Province.

WORLDBANK 2015. Adolescent fertility rate (births per 1,000 women ages 15-19).

# APPENDICES

**APPENDIX I: CONSENT FORM FOR ADULT PARTICIPANTS (18 – 19 YEARS)**

MAKERERE UNIVERSITY

SCHOOL OF MEDICINE INSTITUTIONAL REVIEW BOARD

(SOM-IRB)

**Title:** Uptake of modern contraceptives among refugee adolescents in Northern Uganda: associated factors, adherence, lived experiences and effect of peer counselling.

**Investigator:** Bakesiima Ritah (BLT, MSc), Makerere University, College of Health Sciences, Department of Obstetrics and Gynaecology, Tel No; 0772 029182, 0706 807057

**Supervisors:** Dr. Jolly Beyeza-Kashesya, Dr. Rose Nabirye Chalo, Dr. Elin Larsson and Prof. Kristina Gemzell-Danielsson

**Introduction**

You are kindly requested to participate in this study aiming at determining the uptake of modern contraceptives among refugee adolescents in Northern Uganda, the associated factors, adherence, lived experiences and effect of peer counselling.

**Study Purpose**

Adolescent pregnancies among refugees have been reported to be on the rise because of the continued low uptake of contraceptives among the refugee adolescents. Many refugee adolescents are not using modern contraceptives because of limited knowledge and the side effects of these contraceptives. This study seeks to determine the prevalence of modern contraceptive use among refugee adolescents and the factors that determine the use. This study also aims to determine the effect of peer counselling on uptake of modern contraceptives and the lived experiences of refugee adolescents on modern contraceptives.

**Study procedure**

If you agree to participate in this study, you will be asked a few questions regarding your social life as well as your sexual and reproductive history. All the information obtained will be kept confidential.

**Confidentiality**

All the information about you will be kept confidential by the use of identification codes instead of your names. All the forms and papers will be kept under lock and key and other information on the computers will be secured with passwords. No names or initials of any study participants will be used in any publications from this study.

**Benefits of participating**

You will not receive any direct benefits, however the information obtained from this study will be used to help improve adolescent contraceptive use which will in the long run help to reduce adolescent pregnancies.

**Risks / inconveniences**

No risks are associated with participating in this study, and no samples will be taken from your body. You will however be asked to give us 15 – 20 minutes of your time and may experience some discomfort answering some of the questions. This will however not be beyond what is normally experienced during an interview.

**Rights to decline or withdraw from this study**

Your participation in this study is completely voluntary, so you can choose to decline to participate, or even withdraw from it at any time for any reasons. This will not have any effect on you in any way. You also have a right to choose to or not to answer some of the questions asked.

**Compensation for participating**

To compensate you for your time, you will be given an amount of UGX 10,000 but you will not be paid for participating in the study.

**Whom to contact**

Incase you have any questions, concerns, or complaints as regards this study, you can contact any of the Principal Investigator Bakesiima Ritah on Tel. 0706 807057 and incase you have any queries concerning ethical issues, you may contact Prof. Ponsiano Ocama, Chairman School of Medicine Research and Ethics Committee on Tel. 0414 530020 or the Uganda National Council of Science and Technology (UNCST) on Tel. 0772 404970 or 0414-250431

**Statement of consent**

I confirm that the investigator has read and explained to me all the information about this study, and I have been given the opportunity to ask any questions about it, and my participation. I voluntarily agree to participate in this study and I can withdraw at any time for any reasons without any consequences. Procedures regarding confidentiality have been explained to me as well as the use of these study findings in publications. I also understand that by signing I do not waive any of my legal rights but merely indicate my willingness to participate in this study. I will also be given a copy of the signed consent form.

Participant’s name: ......................................................................

Signature: ................................................................................... Date: ..........................

Interviewer: ...................................................................................

Signature: ..................................................................................... Date: ..........................

APPENDIX II: CONSENT FORM FOR PARENTS/GUARDIANS

MAKERERE UNIVERSITY

SCHOOL OF MEDICINE INSTITUTIONAL REVIEW BOARD

(SOM-IRB)

**Title:** Uptake of modern contraceptives among refugee adolescents in Northern Uganda: associated factors, adherence, lived experiences and effect of peer counselling.

**Investigator:** Bakesiima Ritah (BLT, MSc), Makerere University, College of Health Sciences, Department of Obstetrics and Gynaecology, Tel. 0706 807057

**Supervisors:** Dr. Jolly Beyeza-Kashesya, Dr. Rose Nabirye Chalo, Dr. Elin Larsson and Prof. Kristina Gemzell-Danielsson

**Introduction**

You are kindly requested to allow your daughter/girl under your care to participate in this study aiming at determining the uptake of modern contraceptives among refugee adolescents in Northern Uganda, the associated factors, adherence, lived experiences and effect of peer counselling.

**Study Purpose**

Adolescent pregnancies among refugees have been reported to be on the rise because of the continued low uptake of contraceptives among the refugee adolescents. Many refugee adolescents are not using modern contraceptives because of limited knowledge and the side effects of these contraceptives. This study seeks to determine the prevalence of modern contraceptive use among refugee adolescents and the factors that determine the use. This study also aims to determine the effect of peer counselling on uptake of modern contraceptives and the lived experiences of refugee adolescents on modern contraceptives.

**Study procedure**

If you allow your child to participate in this study, she will be asked a few questions regarding her social life as well as her sexual and reproductive history. All the information obtained will be kept confidential.

**Confidentiality**

All the information about your child will be kept confidential by the use of identification codes instead of her names. All the forms and papers will be kept under lock and key and other information on the computers will be secured with passwords. No names or initials of any study participants will be used in any publications from this study.

**Benefits of participating**

There are no direct benefits attached to your child’s participation in this study, however the information obtained from this study will be used to help improve adolescent contraceptive use which will in the long run help to reduce adolescent pregnancies.

**Risks / inconveniences**

No risks are associated with participating in this study, and no samples will be taken from your child’s body. She will however be asked to give us 15 – 20 minutes of her time and may experience some discomfort answering some of the questions. This will however not be beyond what is normally experienced during an interview.

**Rights to decline or withdraw from this study**

Your child’s participation in this study is completely voluntary, so she can choose to decline to participate, or even withdraw from it at any time for any reasons. You may also withdraw your child from the study at any time. This will not have any effect on you or your child in any way. She also has a right to choose to or not to answer some of the questions asked.

**Compensation for participating**

To compensate your child for her time, she will be given an amount of UGX 10,000 but she will not be paid for participating in the study.

**Whom to contact**

Incase you or your child have any questions, concerns, or complaints as regards this study, you can contact any of the Principal Investigator Bakesiima Ritah on Tel. 0706 807057 and incase you have any queries concerning ethical issues, you may contact Prof. Ponsiano Ocama, Chairman School of Medicine Research and Ethics Committee on Tel. 0414 530020 or the Uganda National Council of Science and Technology (UNCST) on Tel. 0772 404970 or 0414-250431

**Statement of consent**

I confirm that the investigator has read and explained to me all the information about this study, and I have been given the opportunity to ask any questions about it, and my child’s participation. I voluntarily allow my child to participate in this study and she can withdraw at any time for any reasons without any consequences. Procedures regarding confidentiality have been explained to me as well as the use of these study findings in publications. I also understand that by signing I do not waive any of my child’s legal rights but merely indicate my willingness to allow her participate in this study. I will also be given a copy of the signed consent form.

Name of child: ......................................................................................................................

Name of parent/guardian: ......................................................................................................

Parent/ Guardian’s signature: ............................................... Date: ....................

**APPENDIX III: ASSENT FORM FOR MINORS (10 – 17 YEARS)**

**MAKERERE UNIVERSITY**

**SCHOOL OF MEDICINE INSTITUTIONAL REVIEW BOARD (SOM-IRB)**

**Title:** Uptake of modern contraceptives among refugee adolescents in Northern Uganda: associated factors, adherence, lived experiences and effect of peer counselling.

**Investigator:** Bakesiima Ritah (BLT, MSc), Makerere University, College of Health Sciences, Department of Obstetrics and Gynaecology, Tel No; 0772 029182, 0706 807057

**Supervisors:** Dr. Jolly Beyeza-Kashesya, Dr. Rose Nabirye Chalo, Dr. Elin Larsson and Prof. Kristina Gemzell-Danielsson

**Introduction**

You are kindly requested to participate in this study aiming at determining the uptake of modern contraceptives among refugee adolescents in Northern Uganda, the associated factors, adherence, lived experiences and effect of peer counselling.

**Study Purpose**

Adolescent pregnancies among refugees have been reported to be on the rise because of the continued low uptake of contraceptives among the refugee adolescents. Many refugee adolescents are not using modern contraceptives because of limited knowledge and the side effects of these contraceptives. This study seeks to determine the prevalence of modern contraceptive use among refugee adolescents and the factors that determine the use. This study also aims to determine the effect of peer counselling on uptake of modern contraceptives and the lived experiences of refugee adolescents on modern contraceptives.

**Study procedure**

If you agree to participate in this study, you will be asked a few questions regarding your social life as well as your sexual and reproductive history. All the information obtained will be kept confidential.

**Confidentiality**

All the information about you will be kept confidential by the use of identification codes instead of your names. All the forms and papers will be kept under lock and key and other information on the computers will be secured with passwords. No names or initials of any study participants will be used in any publications from this study.

**Benefits of participating**

You will not receive any direct benefits, however the information obtained from this study will be used to help improve adolescent contraceptive use which will in the long run help to reduce adolescent pregnancies.

**Risks / inconveniences**

No risks are associated with participating in this study, and no samples will be taken from your body. You will however be asked to give us 15 – 20 minutes of your time and may experience some discomfort answering some of the questions. This will however not be beyond what is normally experienced during an interview.

**Rights to decline or withdraw from this study**

Your participation in this study is completely voluntary, so you can choose to decline to participate, or even withdraw from it at any time for any reasons. This will not have any effect on you in any way. You also have a right to choose to or not to answer some of the questions asked.

**Compensation for participating**

To compensate you for your time, you will be given an amount of UGX 10,000 but you will not be paid for participating in the study.

**Whom to contact**

Incase you have any questions, concerns, or complaints as regards this study, you can contact any of the Principal Investigator Bakesiima Ritah on Tel. 0706 807057 and incase you have any queries concerning ethical issues, you may contact Prof. Ponsiano Ocama, Chairman School of Medicine Research and Ethics Committee on Tel. 0414 530020 or the Uganda National Council of Science and Technology (UNCST) on Tel. 0772 404970 or 0414-250431

**Statement of assent**

I confirm that the investigator has read and explained to me all the information about this study, and I have been given the opportunity to ask any questions about it, and my participation. I voluntarily agree to participate in this study and I can withdraw at any time for any reasons without any consequences. Procedures regarding confidentiality have been explained to me as well as the use of these study findings in publications. I also understand that by signing I do not waive any of my legal rights but merely indicate my willingness to participate in this study. I will also be given a copy of the signed consent form.

Participant’s name: ......................................................................

Signature: ................................................................................... Date: ..........................

Interviewer: ...................................................................................

Signature: ..................................................................................... Date: ..........................

APPENDIX IV: CONSENT FORM FOR ADULTS (NUER TRANSLATION)

MAKERERE UNIVERSITY

SCHOOL OF MEDICINE INSTITUTIONAL REVIEW BOARD

(SOM-IRB)

**Kacie ke gɔɔr bene kɔcdit cɔk nɔŋ nyic de ke be loi (run ke thiɛr ku bɛr kene thiɛr ku dhoŋuan)**

**Title/Ke be j**ä**mic**: Luï dë ka kë gel dë lïc/ka yene ke dhïth gel në kam dë rïnythi tɔ keke ya abakɔk në lɔŋ cuïc dë Yuganda/northern Uganda: pïanhden ka rëcden, Luïden në nyindhïɛ, ke nyic raan ce ke kan Luɔï në pïïr de yic, ku pïath ka rëc de jiɛm de rïnythi ne kake gel de lïc.

**Investigator/Raan ŋɔr luɔi:** Bakesiima Ritah (BLT, MSc), Makerere University, College of Health Sciences, (Department of Obstetrics and Gynaecology)/akunhöm de diär liäc agut ci dhith, Tel No; 0772 029182, 0706 807057

**Supervisors/Atiɛtnyin:** Dr. Jolly Beyeza-Kashesya, Dr. Rose Nabirye Chalo, Dr. Elin Larsson ku Prof. Kristina Gemzell-Danielsson.

**Introduction/Gɔc**

Yin liɛm ba jäm ne ye jam dïene yic, acan akar ba nyic, ye rïnythi tɔ keke ya abakök kake gel de lïc luɔi? ku jɔl ya pïanhden ka rëcden, Luïdën ne nyindhïɛ, ku ke nyic raan cë ke kan Luɔï në pïïr de yic, ku pïath ka rëc de jiɛm de rïnythi ne biäk de ke kake.

**Study Purpose/luɔi de kɔr ɣe nyinye**

Lïny de nyïrthi tɔ keke ya abakök ee rɔt ya loi në nyindhiɛ ne ke ce rïnythi ye löm ne kake gel de lïc. Abakök juïc ke rïnythi ace löm ne kake gel de lic ne ke cin keek nyic dën kurɛc wen ye tuɔl në luïdën yic. Akar ba nyic ye rïnythi ya abakök kake gel de lïc luöi ku jɔl ya pïanhden ka rëcden, Luïdën ne nyindhïɛ, ke nyic raan cë ke kan Luɔ̈i, ku piäth ka rëc de jiɛm de rïnythi. Akar ba nyic te ye rïnythi ya abakök kake gel de lïc luɔi ne nyindhïɛ ku ka wen ye keek cɔk luï ee ke. Akar ba piäth ka rëc de jiɛm de rïnythi ne biäk de Luï de ke kake nyic, ku ke nyic ke ne lɔŋ de rïnythi ce ke kan Luɔ̈i.

**Study procedure/Dhel de kɔr ɣe nyinye**

Te cin ye gam ba jäm ne ye kene yic, ke yin be thiec ne thual ne lɔŋ de piirduic ku jɔl ya biäk de dhïth. Ka cake luel ebën abe ke tɔu te cene ke be kan yok.

**Confidentiality/Gel bene rin gel**

Ka ba ke luel ne guɔpdu eben abe tɔ ne kam du wene raan bi thic ku ka be ke tɔu te cene ke be kan yok ne randɛ ku rinku acin te beneke gɔɔr. Waragɛk kedhiɛ abe taau te ye thiekthok nɔŋ atuër, ku kɔk tɔne komputai yic abe gel. Acïn rin be ke täu ne ka bake luel thok, ɣago kɔc ee ke luelke ce nyic.

**Benefits of participating/Riöp ba yok ne ye kene yic**

Acin ariöp be yien yin, e nyiny be yok ee ka bene luoide kake gel de lic cök pïny ne kam de rïnythi ɣago linyde nyirthi eyic tek.

**Risks / inconveniences/Ka ce piɛth bi ŋɛi yok**

Acïnke rɛc bi yök ne ye jamɛ yic ku ka cinke thiak ke guɔp be loom. Ee tha thin kor cït degekai ke thier ku dhïc ka thierou ku jɔl ya thuel wen be yi ŋɛɛi riac guɔp. ɣa ce thuel wen räc, ee riɔc de guɔp wen ne ran jamke kaman.

**Rights to decline or withdraw from this study/Yic leu bin jal ne ye kene yic**

Tɔdu ne ye jamɛ yicɣabë ya gem ee rɔtdu. ku yin nɔŋ riɛr lëu bin jal thïn në thaŋdun kɔr te nɔŋ ɣen ke cïn yen bër kɔr. Acïnke rɛc bi loi te jel yin. Yin laau ba thuel kɔk wan keke ci thiöŋ te cin ye täk.

**Compensation for participating/Aleecdu**

Yin be leec ne alipke dhic ke Uganda ne tha dun ca gäm ku ka ce ariop.

**Whom to contact/Raan leu be thiec**

Te nɔŋ yen ke dak yi piɔu ne ye luɔi ee yic, ke yin lëu ba ye raan ŋɔr ee luɔi ɣe cɔl, Bakesiima Ritah on Tel. 0706 807057, ku na nɔŋ ke kɔr ba nyic ne lɔŋ de loŋ ke ye kenne, ke yin lëu ba ye raan ne cɔl, Prof. Ponsiano Ocama, Chairman School of Medicine Research and Ethics Committee on Tel. 0414 530020 or the Uganda National Council of Science and Technology (UNCST) on Tel. 0772 404970 or 0414-250431.

**Statement of consent/cat de gäm**

ɣaya gam raan yen ne be ɣɛn thic thok, ee keriɛc eben be jämic kuën ku juiric apiɛth ku ke ɣɛn yiɛn thäk ban kede ɣapiou thic ne lɔŋ de ke banjäm thïn. ɣɛn ce rɔt gam ba tɔ ne ye luɔi ɣeyic ku ɣɛn nɔŋ riɛr ba jal thin ke cin ke yok ɣɛn. Tɔɔu de ka bake luël ebën agut ci luöi den erɔt nyɛi juïr apiɛth. Aca det nan adë ke thäny cän ɣa cin thäny ekede nyooth can rɔt gäm, ku ka lëu be yïcdië nyaai. ɣɛn be yiɛn töŋ dë waragɛk cake thäny.

Rinku: ......................................................................

Thany yi cin: ................................................................................... pei nin: ..........................

Rinke ran bi thiec: ...................................................................................

Thany de ye cin: .....................................................................................

pei nin: ..........................

APPENDIX V: CONSENT FORM FOR PARENTS/GUARDIANS (NUER TRANSLATION)

MAKERERE UNIVERSITY

SCHOOL OF MEDICINE INSTITUTIONAL REVIEW BOARD

(SOM-IRB)

**Title/Ke be j**ä**mic**: Luï dë ka kë gel dë lïc/ka yene ke dhïth gel në kam dë rïnythi tɔ keke ya abakɔk në lɔŋ cuïc dë Yuganda/northern Uganda: pïanhden ka rëcden, Luïden në nyindhïɛ, ke nyic raan ce ke kan Luɔï në pïïr de yic, ku pïath ka rëc de jiɛm de rïnythi ne kake gel de lïc.

**Investigator/Raan ŋɔr luɔi:** Bakesiima Ritah (BLT, MSc), Makerere University, College of Health Sciences, (Department of Obstetrics and Gynaecology)/akunhöm de diär liäc agut ci dhith, Tel No; 0772 029182, 0706 807057

**Supervisors/Atiɛtnyin:** Dr. Jolly Beyeza-Kashesya, Dr. Rose Nabirye Chalo, Dr. Elin Larsson ku Prof. Kristina Gemzell-Danielsson.

**Introduction/Gɔc**

Yin liɛm ba nyan dun tɔ kene yin pal ɣɛn be tɔ ne ye jam dïene yic, ye rïnythi tɔ keke ya abakök kake gel de lïc luɔi? ku jɔl ya pïanhden ka rɛcden, Luïdën ne nyindhïɛ, ku ke nyic raan cë ke kan luɔi në pïïr de yic, ku pïath ka rɛc de jiɛm de rïnythi ne biäk de ke kake.

**Study Purpose/luɔi de kɔr ɣe nyinye**

Lïny de nyïrthi tɔ keke ya abakök e rɔt ya loi në nyindhiɛ ne ke ce rïnythi ye löm ne kake gel de lïc. Abakök juïc ke rïnythi ɣace löm ne kake gel de lic ne ke cin keek nyic dën kurɛc wen ye tuɔl në luïdën yic. Akar ba nyic ye rïnythi ya abakök kake gel de lïc luɔi ku jɔl ya pïanhden ka rɛcden, luïdën ne nyindhïɛ, ke nyic raan cë ke kan luɔ̈i, ku piäth ka rëc de jiɛm de rïnythi. Akar ba nyic na ye rïnythi ya abakök kake gel de lïc luɔi ne nyindhïɛ ku ka wen ye keek cɔk luï eke. Akar ba piäth ka rɛc de jiɛm de rïnythi ne biäk de Luï de kake nyic, ku ke nyic ke ne lɔŋ de rïnythi ce ke kan Luɔ̈i.

**Study procedure/ Dhol de kɔr ɣe nyinye**

Te pɛle yin ɣa nyandu be jam ne yekene yic, keka be thiec ne thual ne lɔŋ de piirde yic ku jɔl ya biäk de kake dhithic. Ka ceke luel ebën abe tɔu te cene ke be kan yok.

**Confidentiality/Gel bene rin gel**

Kaŋ kedhiɛ kake menhdu, abe tɔ ne kamde kene raan bɛ thiec ku ka be ke tɔu te cene ke be kan yok ne rande ku rinke acin te beneke gɔɔr ago cie nyic. Waragɛk kedhiɛ abe taau te ye thiekthok nɔŋ atuër, ku kɔk tɔne komputai yic abe gel. Acïn rin be ke täu ne ka bake luel thok, ago kɔc eke luelke ce nyic.

**Benefits of participating/Riöp ba yok ne ye kene yic**

Acin ariöp be yien ɣen, ee nyiny be yok eka bene luoide kake gel de lic cök pïny ne kam de rïnythi ago linyde nyirthi eeyic tek.

**Risks / inconveniences/ Ka ce piɛth bi ŋɛi yok**

Acïnke rec bi menhdu yök ne ye jamɛ yic ku ka cinke thiak ke guɔpde menhdu be loom. Ee tha thin kor cït degekai ke thier ku dhïc ka thierou ku jɔl ya thuel wen be yen ŋɛɛi riac guɔp. Ace thuel wen räc, ee riɔc de guɔp wen ne ran jamke kaman.

**Rights to decline or withdraw from this study/ Yic leu bin jal ne ye kene yic**

Tɔ de mäthdu ne ye jamɛ yic abë ya gem ee rɔtde. Ku ka nɔŋ riɛr lëu ben jal thïn në thaŋden kɔr te nɔŋ en ke cen ɣen bër kɔr. Acïnke rec bi luɔi yin ka mɛthdu. Alaau be thuel kɔk wan keke ci thiöŋ te cen ye täk.

**Compensation for participating/**Aleecdu

Mɛthdu abë leec ne alipke dhic ke Uganda ne tha den ce gäm ku ka ce ariop.

**Whom to contact/Raan leu be thiec**

Te nɔŋ yen ke dak yi piɔu ka mɛthdu ne ye luɔi ee yic, ke yin lëu ba ye raan ŋɔr ee luɔi ɣe cɔl, Bakesiima Ritah on Tel. 0706 807057, ku na nɔŋ ke kɔr ba nyic ne lɔŋ de loŋ ke ye kene, ke yin lëu ba ye raane cɔl, Prof. Ponsiano Ocama, Chairman School of Medicine Research and Ethics Committee on Tel. 0414 530020 or the Uganda National Council of Science and Technology (UNCST) on Tel. 0772 404970 or 0414-250431.

**Statement of consent/ cat de gäm**

ɣaya gam raan yen ne be ɣen thic thok, ee keriɛc eben be jämic kuën ku juiric apiɛth ku ke ɣɛn yiɛn thäk ban kede ɣapiou thic ne lɔŋ de ke bene mɛthdie jäm thïn. ɣɛn ce mɛthdie puɔl be tɔ ne ye luɔi ee yic ku ka nɔŋ riɛr ben jal thin ke cin ke yok ɣen. Tɔɔu de ka be ke luël ebën agut ci lui den ee rɔt nyɛi juïr apiɛth. Aca det nan adë ke thäny bän ɣa cin thäny ee kede nyooth can mɛthdie puɔl, ku ka cie be yicde mɛthdie nyaai. ɣen be yiën töŋ dë waragɛk cakke thäny.

Rinke meth:……………………………………………..

Rinke randit de meth: ......................................................................

Thany yi cin: ................................................................................... pɛi nin: ......................

**APPENDIX VI: ASSENT FORM FOR MINORS (NUER TRANSLATION)**

**MAKERERE UNIVERSITY**

**SCHOOL OF MEDICINE INSTITUTIONAL REVIEW BOARD (SOM-IRB)**

**CÄT DE GÄM DE MƐTH (RUN KE THI**Ɛ**R KU DHOROU)**

**Title/Ke be j**ä**mic**: Luï dë ka kë gel dë lïc/ka yene ke dhïth gel në kam dë rïnythi tɔ keke ya abakɔk në lɔŋ cuïc dë Yuganda/northern Uganda: pïanhden ka rëcden, Luïden në nyindhïɛ, ke nyic raan ce ke kan Luɔï në pïïr de yic, ku pïath ka rëc de jiɛm de rïnythi ne kake gel de lïc.

**Investigator/Raan ŋɔr luɔi:** Bakesiima Ritah (BLT, MSc), Makerere University, College of Health Sciences, (Department of Obstetrics and Gynaecology)/akunhöm de diär liäc agut ci dhith, Tel No; 0772 029182, 0706 807057

**Supervisors/Atiɛtnyin:** Dr. Jolly Beyeza-Kashesya, Dr. Rose Nabirye Chalo, Dr. Elin Larsson ku Prof. Kristina Gemzell-Danielsson

**Introduction/Gɔc**

Yin liɛm ba jäm ne ye jam dïene yic, acan akar ba nyic, ye rïnythi tɔ keke ya abakök kake gel de lïc luɔi? ku jɔl ya pïanhden ka rëcden, Luïdën ne nyindhïɛ, ku ke nyic raan cë ke kan Luɔï në pïïr de yic, ku pïath ka rëc de jiɛm de rïnythi ne biäk de ke kake.

**Study Purpose/luɔi de kɔr ɣe nyinye**

Lïny de nyïrthi tɔ keke ya abakök ee rɔt ya loi në nyindhiɛ ne ke ce rïnythi ye löm ne kake gel de lïc. Abakök juïc ke rïnythi ace löm ne kake gel de lic ne ke cin keek nyic dën kurɛc wen ye tuɔl në luïdën yic. Akar ba nyic ye rïnythi ya abakök kake gel de lïc luöi ku jɔl ya pïanhden ka rëcden, Luïdën ne nyindhïɛ, ke nyic raan cë ke kan Luɔ̈i, ku piäth ka rëc de jiɛm de rïnythi. Akar ba nyic te ye rïnythi ya abakök kake gel de lïc luɔi ne nyindhïɛ ku ka wen ye keek cɔk luï ee ke. Akar ba piäth ka rëc de jiɛm de rïnythi ne biäk de Luï de ke kake nyic, ku ke nyic ke ne lɔŋ de rïnythi ce ke kan Luɔ̈i.

**Study procedure/Dhel de kɔr ɣe nyinye**

Te cin ye gam ba jäm ne ye kene yic, ke yin be thiec ne thual ne lɔŋ de piirduic ku jɔl ya biäk de dhïth. Ka cake luel ebën abe ke tɔu te cene ke be kan yok.

**Confidentiality/Gel bene rin gel**

Ka ba ke luel ne guɔpdu eben abe tɔ ne kam du wene raan bi thic ku ka be ke tɔu te cene ke be kan yok ne randɛ ku rinku acin te beneke gɔɔr. Waragɛk kedhiɛ abe taau te ye thiekthok nɔŋ atuër, ku kɔk tɔne komputai yic abe gel. Acïn rin be ke täu ne ka bake luel thok, ɣago kɔc ee ke luelke ce nyic.

**Benefits of participating/Riöp ba yok ne ye kene yic**

Acin ariöp be yien yin, e nyiny be yok ee ka bene luoide kake gel de lic cök pïny ne kam de rïnythi ɣago linyde nyirthi eyic tek.

**Risks / inconveniences/Ka ce piɛth bi ŋɛi yok**

Acïnke rɛc bi yök ne ye jamɛ yic ku ka cinke thiak ke guɔp be loom. Ee tha thin kor cït degekai ke thier ku dhïc ka thierou ku jɔl ya thuel wen be yi ŋɛɛi riac guɔp. ɣa ce thuel wen räc, ee riɔc de guɔp wen ne ran jamke kaman.

**Rights to decline or withdraw from this study/Yic leu bin jal ne ye kene yic**

Tɔdu ne ye jamɛ yicɣabë ya gem ee rɔtdu. ku yin nɔŋ riɛr lëu bin jal thïn në thaŋdun kɔr te nɔŋ ɣen ke cïn yen bër kɔr. Acïnke rɛc bi loi te jel yin. Yin laau ba thuel kɔk wan keke ci thiöŋ te cin ye täk.

**Compensation for participating/Aleecdu**

Yin be leec ne alipke dhic ke Uganda ne tha dun ca gäm ku ka ce ariop.

**Whom to contact/Raan leu be thiec**

Te nɔŋ yen ke dak yi piɔu ne ye luɔi ee yic, ke yin lëu ba ye raan ŋɔr ee luɔi ɣe cɔl, Bakesiima Ritah on Tel. 0706 807057, ku na nɔŋ ke kɔr ba nyic ne lɔŋ de loŋ ke ye kenne, ke yin lëu ba ye raan ne cɔl, Prof. Ponsiano Ocama, Chairman School of Medicine Research and Ethics Committee on Tel. 0414 530020 or the Uganda National Council of Science and Technology (UNCST) on Tel. 0772 404970 or 0414-250431.

**Statement of consent/cat de gäm**

ɣaya gam raan yen ne be ɣɛn thic thok, ee keriɛc eben be jämic kuën ku juiric apiɛth ku ke ɣɛn yiɛn thäk ban kede ɣapiou thic ne lɔŋ de ke ban jäm thïn. ɣɛn ce rɔt gam ba tɔ ne ye luɔi ɣeyic ku ɣɛn nɔŋ riɛr ba jal thin ke cin ke yok ɣɛn. Tɔɔu de ka bake luël ebën agut ci luöi den erɔt nyɛi juïr apiɛth. Aca det nan adë ke thäny cän ɣa cin thäny ekede nyooth can rɔt gäm, ku ka lëu be yïcdië nyaai. ɣɛn be yiɛn töŋ dë waragɛk cake thäny.

Rinku: ......................................................................

Thany yi cin: ................................................................................... pei nin: ..........................

Rinke ran bi thiec: ...................................................................................

Thany de ye cin: ............................................................................pei nin: ..........................

**APPENDIX VII: CONSENT FORM FOR ADULTS (ARABIC TRANSLATION)**

**MAKERERE UNIVERSITY**

**SCHOOL OF MEDICINE INSTITUTIONAL REVIEW BOARD (SOM-IRB)**

***استمارة الموافقة على المشاركين البالغين* (18 - 19 سنة)**

*العنوان* : استخدامموانعالحملالحديثةبينالمراهقيناللاجئينفيشمالأوغندا: العواملالمرتبطةبها،والالتزام،والتجاربالمعيشية،وتأثيرالمشورةمنالأقران.

*المحقق* : BakesiimaRitah (BLT، MSc)،جامعةماكيريري،كليةالعلومالصحية،قسمالتوليدوأمراضالنساء،Tel No؛ 0772 029182 ، 0706 807057

*المشرفون* : د. جوليبييزا-كاشيسيا،د. روزنابيرىشالو،د. إلينلارسونوالبروفيسوركريستيناجيمزيلدانيلسون

*المقدمة*

يرجىالتكرمبالمشاركةفيهذهالدراسةالتيتهدفإلىتحديداستيعابموانعالحملالحديثةبينالمراهقيناللاجئينفيشمالأوغنداوالعواملالمرتبطةبهاوالالتزاموالتجاربالمعيشيةوتأثيرالمشورةمنالأقران.

*دراسةالغرض*

وقدأفيدبأنحالاتالحملبينالمراهقينبيناللاجئينآخذةفيالارتفاعبسبباستمرارانخفاضامتصاصوسائلمنعالحملبينالمراهقيناللاجئين. كثيرمنالمراهقيناللاجئينلايستخدمونوسائلمنعالحملالحديثةبسببمحدوديةالمعرفةوالآثارالجانبيةلموانعالحملهذه. تسعىهذهالدراسةلتحديدمدىانتشاراستخداموسائلمنعالحملالحديثةبينالمراهقيناللاجئينوالعواملالتيتحدداستخدام. تهدفهذهالدراسةأيضاإلىتحديدتأثيرالمشورةمنالأقرانعلىاستيعابموانعالحملالحديثةوالخبراتالحيةللمراهقيناللاجئينعلىوسائلمنعالحملالحديثة.

*إجراءاتالدراسة*

إذاوافقتعلىالمشاركةفيهذهالدراسة،فسوفيُطلبمنكبعضالأسئلةبخصوصحياتكالاجتماعيةبالإضافةإلىتاريخكالجنسيوالإنجابي. سيتمالحفاظعلىسريةجميعالمعلوماتالتيتمالحصولعليها.

*سرية*

سيتمالحفاظعلىسريةجميعالمعلوماتعنكباستخدامرموزالتعريفبدلاًمنأسماءك. سيتمالاحتفاظبجميعالنماذجوالأوراقتحتقفلومفتاحوسيتمتأمينمعلوماتأخرىعلىأجهزةالكمبيوترمعكلماتالمرور. لنيتماستخدامأيأسماءأوأحرفأوليةمنأيمشاركفيالدراسةفيأيمنشوراتمنهذهالدراسة.

*فوائدالمشاركة*

لنتتلقىأيفوائدمباشرة،ولكنسيتماستخدامالمعلوماتالتيتمالحصولعليهامنهذهالدراسةللمساعدةفيتحسيناستخداموسائلمنعالحملللمراهقينوالتيستساعدعلىالمدىالطويلعلىتقليلحالاتالحملبينالمراهقات.

*المخاطر / المضايقات*

لاتوجدمخاطرمرتبطةبالمشاركةفيهذهالدراسة،ولنيتمأخذعيناتمنجسمك. ومعذلك،سيُطلبمنكتقديم 15 إلى 20 دقيقةمنوقتكوقدتواجهبعضالانزعاجفيالإجابةعنبعضالأسئلة. ولكنهذالنيتجاوزمايتماختبارهعادةخلالالمقابلة.

حقوقالرفضأوالانسحابمنهذهالدراسة

تعتبرمشاركتكفيهذهالدراسةاختياريةتمامًا،لذايمكنكاختياررفضالمشاركةأوحتىالانسحابمنهافيأيوقتولأيسبب. هذالنيكونلهأيتأثيرعليكبأيشكلمنالأشكال. لديكأيضًاالحقفياختيارالإجابةعنبعضالأسئلةأوعدمالردعليها.

*التعويضعنالمشاركة*

لتعويضكعنوقتك،سيتمإعطاؤكمبلغًاقدره 10000 UGX ولكنلنيتمالدفعلكنظيرمشاركتكفيالدراسة.

*لمنالاتصال*

إذاكانلديكأيأسئلةأومخاوفأوشكاوىفيمايتعلقبهذهالدراسة،يمكنكالاتصالبأيمنالباحثالرئيسيBakesiimaRitahعلىالهاتف. 0706 807057 وإذاكانلديكأياستفساراتتتعلقبالقضاياالأخلاقية،فيمكنكالاتصالبـProf. PonsianoOcama،رئيسلجنةالبحثفيالطبولجنةالأخلاقياتعلىالهاتف. 0414 530020أوالمجلسالوطنيالأوغنديللعلوموالتكنولوجيا (UNCST) علىالهاتف. 0772 404970 أو 0414-250431

*بيانالموافقة*

أؤكدأنالمحقققدقرأوشرحليجميعالمعلوماتحولهذهالدراسة،وقدأتيحتليالفرصةلطرحأيأسئلةحولها،ومشاركتي. أوافقطواعيةعلىالمشاركةفيهذهالدراسةويمكننيالانسحابفيأيوقتولأيسببدونأيعواقب. لقدتمشرحالإجراءاتالمتعلقةبالسريةبالإضافةإلىاستخدامنتائجالدراسةفيالمنشورات. أفهمأيضًاأنهمنخلالالتوقيع،لاأتنازلعنأيمنحقوقيالقانونيةولكنيأشيرفقطإلىرغبتيفيالمشاركةفيهذهالدراسة. سأحصلأيضاعلىنسخةمناستمارةالموافقةالموقعة.

اسمالمشترك: ............................................... .......................

التوقيع: ................................................ ................................... تاريخ: ............. .............

الباحث: ................................................ ...................................

التوقيع: ................................................ ..................................... تاريخ: ........... ...............

**APPENDIX VIII: CONSENT FORM FOR PARENTS/GUARDIANS (ARABIC TRANSLATION)**

**MAKERERE UNIVERSITY**

**SCHOOL OF MEDICINE INSTITUTIONAL REVIEW BOARD (SOM-IRB)**

استمارةالموافقةعلىالوالدين / الحراس

**العنوان*:

استخدامموانعالحملالحديثةبينالمراهقيناللاجئينفيشمالأوغندا: العواملالمرتبطةبها،والالتزام،والتجاربالمعيشية،وتأثيرالمشورةمنالأقران.

**الباحث:*

Bakesiima Ritah (BLT، MSc)،جامعةماكيريري،كليةالعلومالصحية،قسمالتوليدوأمراضالنساء،Tel. 0706 807057

المشرفون: د. جوليبييزا-كاشيسيا،د. روزنابيرىشالو،د. إلينلارسونوالبروفيسوركريستيناجيمزيلدانيلسون

*المقدمة*

يرجىالتكرمبالسماحلابنتك / ابنتكتحترعايتكبالمشاركةفيهذهالدراسةالتيتهدفإلىتحديداستيعابموانعالحملالحديثةبينالمراهقيناللاجئينفيشمالأوغنداوالعواملالمرتبطةبهاوالالتزاموالتجاربالمعيشيةوتأثيرالمشورةمنالأقران.

*دراسةالغرض*

وقدأفيدبأنحالاتالحملبينالمراهقينبيناللاجئينآخذةفيالارتفاعبسبباستمرارانخفاضامتصاصوسائلمنعالحملبينالمراهقيناللاجئين. كثيرمنالمراهقيناللاجئينلايستخدمونوسائلمنعالحملالحديثةبسببمحدوديةالمعرفةوالآثارالجانبيةلموانعالحملهذه. تسعىهذهالدراسةلتحديدمدىانتشاراستخداموسائلمنعالحملالحديثةبينالمراهقيناللاجئينوالعواملالتيتحدداستخدام. تهدفهذهالدراسةأيضاإلىتحديدتأثيرالمشورةمنالأقرانعلىاستيعابموانعالحملالحديثةوالخبراتالحيةللمراهقيناللاجئينعلىوسائلمنعالحملالحديثة.

*إجراءاتالدراسة*

إذاسمحتلطفلكبالمشاركةفيهذهالدراسة،فسوفيُسألعنهابعضالأسئلةالمتعلقةبحياتهاالاجتماعيةبالإضافةإلىتاريخهاالجنسيوالإنجابي. سيتمالحفاظعلىسريةجميعالمعلوماتالتيتمالحصولعليها.

*سرية*

سيتمالحفاظعلىسريةجميعالمعلوماتالمتعلقةبطفلكعنطريقاستخدامرموزالتعريفبدلاًمنأسمائها. سيتمالاحتفاظبجميعالنماذجوالأوراقتحتقفلومفتاحوسيتمتأمينمعلوماتأخرىعلىأجهزةالكمبيوترمعكلماتالمرور. لنيتماستخدامأيأسماءأوأحرفأوليةمنأيمشاركفيالدراسةفيأيمنشوراتمنهذهالدراسة.

*فوائدالمشاركة*

لاتوجدفوائدمباشرةمرتبطةبمشاركةطفلكفيهذهالدراسة،ولكنسيتماستخدامالمعلوماتالتيتمالحصولعليهامنهذهالدراسةللمساعدةفيتحسيناستخداموسائلمنعالحملللمراهقينوالتيستساعدعلىالمدىالطويلعلىتقليلحالاتالحمللدىالمراهقين.

*المخاطر / المضايقات*

لاتوجدمخاطرمرتبطةبالمشاركةفيهذهالدراسة،ولنيتمأخذأيعيناتمنجسمطفلك. ومعذلك،سيُطلبمنهاإعطاؤنا 15-20 دقيقةمنوقتهاوقدتواجهبعضالانزعاجفيالإجابةعنبعضالأسئلة. ولكنهذالنيتجاوزمايتماختبارهعادةخلالالمقابلة.

*حقوقالرفضأوالانسحابمنهذهالدراسة*

تعتبرمشاركةطفلكفيهذهالدراسةاختياريةتمامًا،لذايمكنهااختياررفضالمشاركةأوالانسحابمنهافيأيوقتولأيسبب. يمكنكأيضًاسحبطفلكمنالدراسةفيأيوقت. هذالنيكونلهأيتأثيرعليكأوعلىطفلكبأيشكلمنالأشكال. كمايحقلهااختيارالإجابةعنبعضالأسئلةأوعدمالردعليها.

*التعويضعنالمشاركة*

لتعويضطفلكعنوقتها،سيتمإعطاؤهمبلغًاقدره 10000 UGX ،لكنهالنتحصلعلىأجرمقابلمشاركتهافيالدراسة.

*لمنالاتصال*

فيحالكانلديكأولطفلكأيأسئلةأومخاوفأوشكاوىفيمايتعلقبهذهالدراسة،يمكنكالاتصالبأيمنالباحثالرئيسيBakesiimaRitahعلىالهاتف. 0706807057 وإذاكانلديكأياستفساراتتتعلقبالقضاياالأخلاقية،فيمكنكالاتصالبـProf. PonsianoOcama،رئيسلجنةالبحثفيالطبولجنةالأخلاقياتعلىالهاتف. 0414 530020 أوالمجلسالوطنيالأوغنديللعلوموالتكنولوجيا (UNCST) علىالهاتف. 0772 404970 أو 0414-250431

*بيانالموافقة*

أؤكدأنالمحقققدقرأوشرحليجميعالمعلوماتحولهذهالدراسة،وقدأتيحتليالفرصةلطرحأيأسئلةحولها،ومشاركةطفلي. أسمحطواعيةلطفليبالمشاركةفيهذهالدراسةويمكنهاالانسحابفيأيوقتولأيسببدونأيعواقب. لقدتمشرحالإجراءاتالمتعلقةبالسريةبالإضافةإلىاستخدامنتائجالدراسةفيالمنشورات. أفهمأيضًاأنهمنخلالالتوقيع،لاأتنازلعنأيحقوققانونيةلطفلي،ولكنيأشيرفقطإلىرغبتيفيالسماحلهابالمشاركةفيهذهالدراسة. سأحصلأيضاعلىنسخةمناستمارةالموافقةالموقعة.

اسمالطفل: .............................................. .................................................. ......................

اسمالوالد / الوصي: ............................................ .................................................. ........

توقيعوليالأمر / الوصي: ............................................. .. تاريخ: ...................

**APPENDIX IX: ASSENT FORM FOR MINORS (ARABIC TRANSLATION)**

**MAKERERE UNIVERSITY**

**SCHOOL OF MEDICINE INSTITUTIONAL REVIEW BOARD (SOM-IRB)**

*العنوان* :

استخدامموانعالحملالحديثةبينالمراهقيناللاجئينفيشمالأوغندا: العواملالمرتبطةبها،والالتزام،والتجاربالمعيشية،وتأثيرالمشورةمنالأقران.

*المحقق* :

Bakesiima Ritah (BLT، MSc)،جامعةماكيريري،كليةالعلومالصحية،قسمالتوليدوأمراضالنساء،Tel No؛ 0772 029182 ، 0706 807057

*المشرفون* :

د. جوليبييزا-كاشيسيا،د. روزنابيرىشالو،د. إلينلارسونوالبروفيسوركريستيناجيمزيلدانيلسون

*المقدمة*

يرجىالتكرمبالمشاركةفيهذهالدراسةالتيتهدفإلىتحديداستيعابموانعالحملالحديثةبينالمراهقيناللاجئينفيشمالأوغنداوالعواملالمرتبطةبهاوالالتزاموالتجاربالمعيشيةوتأثيرالمشورةمنالأقران.

*دراسةالغرض*

وقدأفيدبأنحالاتالحملبينالمراهقينبيناللاجئينآخذةفيالارتفاعبسبباستمرارانخفاضامتصاصوسائلمنعالحملبينالمراهقيناللاجئين. كثيرمنالمراهقيناللاجئينلايستخدمونوسائلمنعالحملالحديثةبسببمحدوديةالمعرفةوالآثارالجانبيةلموانعالحملهذه. تسعىهذهالدراسةلتحديدمدىانتشاراستخداموسائلمنعالحملالحديثةبينالمراهقيناللاجئينوالعواملالتيتحدداستخدام. تهدفهذهالدراسةأيضاإلىتحديدتأثيرالمشورةمنالأقرانعلىاستيعابموانعالحملالحديثةوالخبراتالحيةللمراهقيناللاجئينعلىوسائلمنعالحملالحديثة.

*إجراءاتالدراسة*

إذاوافقتعلىالمشاركةفيهذهالدراسة،فسوفيُطلبمنكبعضالأسئلةبخصوصحياتكالاجتماعيةبالإضافةإلىتاريخكالجنسيوالإنجابي. سيتمالحفاظعلىسريةجميعالمعلوماتالتيتمالحصولعليها.

*سرية*

سيتمالحفاظعلىسريةجميعالمعلوماتعنكباستخدامرموزالتعريفبدلاًمنأسماءك. سيتمالاحتفاظبجميعالنماذجوالأوراقتحتقفلومفتاحوسيتمتأمينمعلوماتأخرىعلىأجهزةالكمبيوترمعكلماتالمرور. لنيتماستخدامأيأسماءأوأحرفأوليةمنأيمشاركفيالدراسةفيأيمنشوراتمنهذهالدراسة.

*فوائدالمشاركة*

لنتتلقىأيفوائدمباشرة،ولكنسيتماستخدامالمعلوماتالتيتمالحصولعليهامنهذهالدراسةللمساعدةفيتحسيناستخداموسائلمنعالحملللمراهقينوالتيستساعدعلىالمدىالطويلعلىتقليلحالاتالحملبينالمراهقات.

*المخاطر / المضايقات*

لاتوجدمخاطرمرتبطةبالمشاركةفيهذهالدراسة،ولنيتمأخذعيناتمنجسمك. ومعذلك،سيُطلبمنكتقديم 15 إلى 20 دقيقةمنوقتكوقدتواجهبعضالانزعاجفيالإجابةعنبعضالأسئلة. ولكنهذالنيتجاوزمايتماختبارهعادةخلالالمقابلة.

*حقوقالرفضأوالانسحابمنهذهالدراسة*

تعتبرمشاركتكفيهذهالدراسةاختياريةتمامًا،لذايمكنكاختياررفضالمشاركةأوحتىالانسحابمنهافيأيوقتولأيسبب. هذالنيكونلهأيتأثيرعليكبأيشكلمنالأشكال. لديكأيضًاالحقفياختيارالإجابةعنبعضالأسئلةأوعدمالردعليها.

*التعويضعنالمشاركة*

لتعويضكعنوقتك،سيتمإعطاؤكمبلغًاقدره 10000 UGX ولكنلنيتمالدفعلكنظيرمشاركتكفيالدراسة.

*لمنالاتصال*

إذاكانلديكأيأسئلةأومخاوفأوشكاوىفيمايتعلقبهذهالدراسة،يمكنكالاتصالبأيمنالباحثالرئيسيBakesiima Ritahعلىالهاتف. 0706 807057 وإذاكانلديكأياستفساراتتتعلقبالقضاياالأخلاقية،فيمكنكالاتصالبـProf. Ponsiano Ocama،رئيسلجنةالبحثفيالطبولجنةالأخلاقياتعلىالهاتف. 0414 530020 أوالمجلسالوطنيالأوغنديللعلوموالتكنولوجيا (UNCST) علىالهاتف. 0772 404970 أو 0414-250431

*بيانالموافقة*

أؤكدأنالمحقققدقرأوشرحليجميعالمعلوماتحولهذهالدراسة،وقدأتيحتليالفرصةلطرحأيأسئلةحولها،ومشاركتي. أوافقطواعيةعلىالمشاركةفيهذهالدراسةويمكننيالانسحابفيأيوقتولأيسببدونأيعواقب. لقدتمشرحالإجراءاتالمتعلقةبالسريةبالإضافةإلىاستخدامنتائجالدراسةفيالمنشورات. أفهمأيضًاأنهمنخلالالتوقيع،لاأتنازلعنأيمنحقوقيالقانونيةولكنيأشيرفقطإلىرغبتيفيالمشاركةفيهذهالدراسة. سأحصلأيضاعلىنسخةمناستمارةالموافقةالموقعة.

اسمالمشترك: ............................................... .......................

التوقيع: ................................................ ................................... تاريخ: ............. .............

الباحث: ................................................ ...................................

التوقيع: ................................................ ..................................... تاريخ: ........... ...............

**APPENDIX X: QUESTIONNAIRE**

**A QUESTIONNAIRE TO STUDY UPTAKE OF MODERN CONTRACEPTIVES AMONG REFUGEE ADOLESCENTS IN NORTHERN UGANDA**

**Participant ID Number:** ................................... **Date of Interview:** ..................

**Social Demographics**

1. What is your age from the last birthday? Age in completed years: ...........
2. What is your religion? Catholic 1

Anglican 2

Moslem 3

Pentecostal 4

Seventh day Adventist 5

Other (Specify)....................... 6

1. What is the highest level of education you None 1

have attained? Primary 2

Secondary (O level) 3

High school (A level) 4

Tertiary 5

1. What ethnic group do you belong to? Acholi 1

Dinka 2

Nuer 3

Other (Specify)...................... 4

1. What is your occupation (what kind of work Self employed 1

do you do)? Employed 2

Unemployed 3

Peasant farmer 4

Student 5

1. How long have you lived in the camp? Less than 6 months 1

6 to 12 months 2

More than a year 3

1. Who are staying with currently? Parent / guardian 1

Friend 2

Partner (husband/boyfriend) 3

Alone 4

1. What is your marital status? Single 1

Currently married 2

Living together/cohabiting 3

Divorced/separated 4

Widowed 5

1. a) What type of union are you in? Monogamous 1

Polygamous 2

1. How long have you been in this union? _______Years/______Months

**Sexual history**

1. Have you ever had sex? Yes 1

No 2

1. If yes, how old were you at first sex? ______________Years
2. How many sexual partners do you have? ­­­­­­­­­­­­­­­­­Number_________________

Do not remember 9

1. How many different partners have you had Number _________________

sexual intercourse with in the past 12 months? Do not remember 9

1. a) Have you ever been given sex education? Yes 1

No 2

b) If yes, what was the source of the sex education? Family 1

Friends 2

School 3

Health worker 4

Other (specify) 5

**Reproductive history**

1. Have you ever been pregnant? Yes 1

No 2

If yes, go to 2. If no, skip to 8

1. How many pregnancies have you ever had? ____________
2. Were any of these pregnancies unintended? Yes 1

No 2

1. What was the cause of the unintended pregnancy? No use of contraception 1

Failed contraceptive 2

Forced or coerced sex 3

Other (specify) ___________ 4

1. If any of the pregnancies was unintended, how Safe abortion(medical/surgical) 1

did you deal with it (especially the most recent)? Unsafe abortion 2

Prepared to give birth 3

Other (specify) ___________ 4

1. How many live births have you ever had? ____________
2. a) How many children do you have alive at the ____________

moment?

b) What are their sexes? Males _______

Females _____

1. How many children do you hope to have in future? ____________

**Spousal information**

1. How old is your spouse? Age in completed years _______
2. What is the highest level of education your Primary 1

spouse has attained? Secondary (O level) 2

High school (A level) 3

Tertiary 4

1. What is your spouse’s occupation? Self employed 1

Employed 2

Unemployed 3

Peasant farmer 4

Student 5

1. How would you rate your communication Very good 1

with your spouse? Good 2

Fair 3

Bad 4

1. How would you rate your partner’s desire to Not at all 1

have children? May be he wants 2

Definitely wants 3

Not sure 4

1. Does spouse’s desire for children influence Yes 1

your desire for children? No 2

**Knowledge on contraceptives**

1. a) Have you ever heard about modern contraceptives? Yes 1

No 2

b) If yes, from where or who did you hear about them? Family 1

Friends 2

School 3

Health worker 4

Media(Radio/T.V) 5

Drama group 6

Poster 7

Other (specify) __________ 8

c) Do you know atleast two modern contraceptives? Yes 1

No 2

1. Who do you think should use modern contraceptives? Married couples only 1

Sexually active people 2

Adults only 3

Anyone who wants 4

Don’t know 5

1. What do you think contraceptives should be used for? Spacing children 1

Limiting children 2

Both 1 and 2 3

Should not be used at all 4

1. Whose role do you think it is to obtain, accept and Men only 1

gain knowledge about contraceptive use? Women only 2

Both 3

**Contraceptive use**

1. Have you ever used any modern contraceptives? Yes 1

No 2

1. Are you currently using any modern contraceptives? Yes 1

No 2

If yes, answer (3) and (4). If no, answer (6).

1. What modern contraceptive method are you currently Condom 1

using? Oral contraceptive (pill) 2

Injectable contraceptive 3

Implant 4

IUD 5

Other (specify) __________ 6

1. How long have you been using the modern _________Years/______Months

Contraceptive mentioned in (3) above?

1. Reasons for not using modern contraceptives Not sexually active 1

Religious prohibitions 2

Cultural prohibitions 3

Partner prohibitions 4

Health reasons 5

Fear of side effects 6

Lack of knowledge 7

Other (specify) ___________ 8

1. Would you consider using any form of modern Yes 1

contraceptives in future? No 2

1. If yes, which one would you consider? Condom 1

Oral contraceptive (pill) 2

Injectable contraceptive 3

Implant 4

IUD 5

Other (specify) ___________ 6

1. What source(s) of modern contraceptives do Health facility 1

you know? Friends 2

Family 3

Other (specify) __________ 4

1. Do you know a contraceptive source in 10 minutes? Yes 1

No 2

***Thank You for Participating***

**APPENDIX XI: QUESTIONNAIRE (NUER TRANSLATION)**

**QUESTIONNAIRE TO STUDY UPTAKE OF MODERN CONTRACEPTIVES AMONG REFUGEE ADOLESCENTS IN NORTHERN UGANDA**

**Numba dune ID:** ................................... **peininthiec yin thok:** ..................

**Social Demographics/kakechiɛŋ de ran**

1. Nɔŋ run ke de? …………............
2. Yi ran ë kanitha ne kekakeyic? Kathilic 1

Yagelikan 2

Muthilim 3

Pentecothol 4

Thebendei 5

Luaŋdetecenekake (luelrin).....6

1. Ca kanlɔdhelɣedene thukulic? ɣɛn ken kan lɔ 1

Peraimeri 2

Thekondari (O level) 3

A lebo (A level) 4

Tertiary/Jama 5

1. Ye jur yin de? Dinka 1

Nuer 2

Other (luelrin)...................... 3

1. Yeŋöye loi piiryin? ɣɛn lui ne rɔt 1

ɣɛn lui 2

acïn te luɔiɣɛn 3

ɣɛnne pur 4

ɣɛnnemɛnhethukul 5

1. Nɔŋ run ka pɛikede ne kemic? Akenpeikedhetem der 1

Atɔ ne kam de pei ke dhetem ke ruɔn 2

Aruɔnbaknhɔm 3

1. Ye ŋaciŋke yinɛmane? Kɔckuɔ/kɔctiɛtnyin ne ɣa 1

Mathdie 2

Monyceɣɛnthiak/mony ë gɔk 3

ɣatok 4

1. Cie yin thiäk? ɣɛnkennekanthiäk 1

ɣɛncethiak 2

ɣɛnrɛrkewɔ ken rɔththiäk 3

Wɔcepuɔ 4

ɣɛnyaabaar 5

1. a) Nɔŋmonydudiarkede? ɣɛnɣatok 1

Wɔjuic 2

b) Canɔŋ run ka peikede ne thiŋduic? _______Run______Pei

**Reproductive history/biakde dhieth**

1. Ca meth kan yok?

Aca kan yok 1

Akenkan yok 2

Na ca meth kan yok,ke yin thioŋthualderou.

Na kene meth kan yok ke yin lɔrɣethualdebiɛr

1. Ca mith yok ɣadɛ? ____________
2. Nɔŋlïcɣekenrɔt juïr ne ke yic? ɣeyic 1 Acieyic 2
3. Ye ŋocɔkyi yok meth kenejuïr? Acin kake gel de lic ɣeke cakke luɔi 1

Kake gel de dhithakeken lui apiɛth 2

ɣɛnneteceɣeriɛr 3

Kɔk (luel rin) 4

1. Ca linyɣeken rɔtjuïr be loiyede? Aca be lɔriɔkpanakem 1

Aca be riɔk bai 2

ɣa ca be tit badhith 3

Tede can ɣenluɔi thin (luel)__4

1. Ye mithkedecakkedhith ɣeke pir? ____________
2. a) Nɔŋmithpiirkedeemane? ____________

b) Yi ke dhuɔl ka nyiir Dhuɔk _______

Nyiir ________

1. Ye ŋɔthke yin be naŋ mith kede ne kolciɛn? ____________

**Spousal information/biak de monydu**

1. Nɔŋmonydu run kede? ______________
2. Ce monyduɣetdhelyïndëɣethukulic? Peraimeri 1 Thekondari 2 A lebo 3 Jama 4

1. Lui monyduteno? Alui ne rɔtde 1

ɣeluɔi 2

ɣakeneluɔi 3

ɣedupur 4

ɣe meth thukul 5

1. Ye jämdɔntɔɣedewenemonydu? Apiɛthapɛi 1

Apiɛth 2

ɣa tom tɔ 3

Acipiɛth 4

1. Ye nhierkɔrɣemonydu be naŋ mith Acienhiar 1

tiŋ wudɛ? Tekdekɔr 2

ɣacubakɔr 3

Aca nyic 4

1. Ye kɔr ye monydumith kɔɔr kɔrdun ɣeyic 1

de mith riɔkic? Acieyic 2

**Sexual history/Biäk de thɔny ke rör**

1. Ca kan tɛc wene moc? ɣeyic 1

ɣaceyic 2

1. Te yen yic, ke yin

nɔŋrunkedeɣeyötuɛŋ du we moc? ______________Run

1. Nɔŋrörkede? Adenden_______________

ɣa ca nyic 9

1. Ca thiak we rörkede ne pëi ce lɔ ke Adenden ________________

thierkurou? ɣa ca nyic 9

1. a) Ci kan piɔɔc ne biak de tɛckerör? ɣeyic 1

ɣaceyic 2

b) Na ye yic,ɣe yin yok piöc de Bai 1

biäk de tɛc ke rör teno? Mɛthcie 2

Thukulic 3

Raanyaakem 4

Kɔk(luelrin) _____________ 5

**Knowl edge on contraceptives/Nyic ne biäk de kake gel de lic**

1. a)Nɔŋ kake gel de lic ca ke kan piŋ? ɣeyic 1

ɣaceyic 2

b) Te yen yic, ye tanŋo ka yeŋaɣe Bai 1

piŋ yin keek? Mɛthcie 2

Thukulic 3

Raanyaakem 4

Radiyo 5

Kɔc ke kereen 6

Athur 7

Kɔk(luelrin) ______________8

1. Nɔŋ kakaaciɛk ke gel de lic Anɔŋ ka nyiɛcke 1

nyicke kerou? No/ Acin ka nyiɛc kek 2

1. Yeŋa ye tiŋ ke piɛth bi kake gel de Kɔcciethïk 1

dhith luɔi? Kɔcnhiartɛɛckeror ka diar 2 Kɔcditkepɛi 3 Ran kɔɔrɣen 4

ɣa ca nyic 5

1. Yeŋö ye tiŋ bene kake gel de Ago mithnaŋkam 1

dhith ya luɔi? Ago mithtekic 2

Ka tuɛŋkerou 3

Aciepiɛth bi keyaluɔi 4

1. Ye yok ci luciɣeŋa bi kake gel de dhithya Rorkepɛi 1

yok, ku gamkekkunyicke? Diarkepɛi 2

Diarkeror 3

**Contraceptive use/Lui de kake gel de lic**

1. Ca kake gel de lic kan luɔi? ɣeyic 1 Acieyic 2
2. Nɔŋkake gel de lic luɔi ɣeke ɛmane? ɣeyic 1

Acieyic 2

Te yen yic, ke yin jopnumbaɣediäkkeŋuan

Tecenɣeyic, ke yin jopnumbaɣedhetem

1. Ye keno dekake gel de licluɔiɣekeɛmane Lithik 1

Wal ye dek 2

Wal ye wuɔ̈m 3

Walye tau ne riŋic 4

Wal ye tau adhiɛthic 5

Kɔk (luelrin) __________ 6

1. Ca naŋ run/pɛikedeke yin luieekede _________Run______Pɛi

gel de liccie?

1. Ka yi gel ne lui de kake gel de lic? ɣɛncietɛɛcwɔror 1

Acieloŋnhialickɔr 2

Acieloŋɣejiɛŋkɔr 3

Aciemonydiekɔr 4

Ka pial de guɔp 5

Riɔc de ka ce piɛth ye tuɔl ne luiden 6

Ciennyic 7

Kɔk (Luelciennyic) ________8

1. De kenɔŋkake gel de licluɔieekeek ɣeyic 1

ne thɛɛk be ben? ɣaceyic 2

1. Te yen yickeyeŋoluɔi ne kekakeyic? Lithik 1

Wal ye dek 2

Walyewuɔ̈m 3

Walye tau ne riŋic 4

Wal ye tau adhiɛthic 5

Kɔk(luelrin) ___________ 6

1. Ye te yïndenyicyenekake gel delicyok? Panakim 1

Mɛth 2

Bai 3

Kɔk(luelrin) _____________ 4

1. Nɔŋtethiɔk de gel de kakelic leu be ɣeyic 1

degɛkai ke thiɛr jot ɣe jal panduɔn/ ɣa ce yic 2

pandu?

***Yin ca lec ca rɔtgäm***

**APPENDIX XII: QUESTIONNAIRE (ARABIC TRANSLATION)**

**A QUESTIONNAIRE TO STUDY UPTAKE OF MODERN CONTRACEPTIVES AMONG REFUGEE ADOLESCENTS IN NORTHERN UGANDA**

تاريخالمقابلة: ....... ........... رقممعرفالمشارك: ..................................

**التركيبةالسكانيةالاجتماعية**

العمرفيالسنواتالمكتملة: ....... ماهوعمركمنعيدالميلادالماضي؟1.

ماهيديانتك؟2.

1. الكاثوليكية

2.انجلكان

3.مسلم

4.بنتكوستول

5. السبتيةاليومالسابع

6.الاخرىحدد......................

ماهوأعلىمستوىتعليملك3.

1.مافي

2. لهاالابتدائي؟

3. الثانوية (مستوىيا)

4. المدرسةالعليا

5.جامعة/معهد

ماهيالمجموعةالعرقيةالتيتنتميإليها؟4.

1.دينك

2.نوير

3. أخرى (حدد) ......................

؟ماهومهنتك (مانوعالعملالذيتقومبه)5.

1. العاملينلحسابهمالخاص

2. مستخدم

3. العاطلينعنالعمل.

4.فلاّح

5.طالب/طالبة

منذمتىوأنتتعيشفيالمخيم؟6.

1. أقلمن 6 أشهر

2. من 6 إلى 12 شهرًا

3. أكثرمنعام

منيقيمونحاليا؟7.

1. الوالد / الوصي

2. صديق

3. شريك (الزوج / صديق)

4. وحده

ماهووضعكالعائلي؟8.

1. لامتزوج

2.متزوج

3. العيشمعا / المعاشرة

4.فصل/طالف

5.ارمل/ارملة

أ) مانوعالاتحادالذيأنتفيه؟9.

1. أحاديالزواج

2.متعدد

______الشهور_______سنوات منذمتىوانتفيهذاالاتح

**التاريخالانجابي**

هلكنتحامل؟1.

1. نعم

2. لا

  إذاكانتالإجابةبنعم،فانتقلإلى 2. إذاكانتالإجابة "لا" ،فانتقلإلى 8

___________ كمعددحالاتالحملالتيمررتبها؟2.

هلكانأيمنحالاتالحملهذهغيرمقصود؟3.

1.نعم

2.لا

ماهوسببالحملغيرالمقصود؟4.

1. عدماستخداموسائلمنعالحمل

2. فابرمانعالحمل

3. الجنسأوقسرا

4. أخرى (حدد) ___________

إذاكانأيمنالحمولغيرمقصود،كيفتعاملتمعهخاصةآخرها؟5.

الطبي.1الإجهاضالآمن

2. الإجهاضغيرالآمن

3. استعدادللولادة

الاخري(حدد).4

_____________________ كمعددالمواليدالأحياءلديك؟6.

____________ أ) كمعددالأطفاللديكعلىقيدالحياةفيالوقتالحالي؟7.

_________   ذكور ب) ماهوجنسهم؟

________    الإناث

______________ ج) كمعددالأطفالالذينتأملفيالحصولعليهافيالمستقبل؟8.

**معلوماتالزوجية**

العمرفيالسنواتالمكتملة ________ كمعمرزوجك؟1.

1.الابتدائية ماهوأعلىمستوىتعليمحصلتعليهزوجتك؟2.

2. الثانوية (مستوىيا)

3. المدرسةالثانوية

4.جامعة/معهد

1. صاحبالعمل ماهيمهنةزوجك؟3.

2.يعمل

3.لايعمل

4. مزارعالمزارعة

5.طالب/طالبة

1. جيدجدا كيفتقيمتواصلكمعزوجك؟4.

2.جيد

3.مقبول

4.ليسجيد

1. لاعلىالإطلاق ماتقييمكلرغبةشريككفيإنجابالأطفال؟5.

2. قديكونيريد

3.يريدعلىالاطلاق

4. بالتأكيد

1.نعم ليرغبزوجكفيالتأثيرعلىالأطفال؟6.

2.لا

**التاريخالجنسي**

1.نعم هلسبقلكأنمارستالجنس؟1.

2.لا

 ______________سنوات إذاكانتالإجابةبنعم،كمكانعمركفيأولممارسةجنسية؟2.

1. رقم ______________ كمعددالشركاءالجنسيينلديك؟3.

2. لاتذكر____________

1. أرقام __________ الجماعالجنسيفيال 12 شهراالماضية؟4.

2. لاتذكر ________

1.نعم أ) هلسبقلكأنحصلتعلىتعليمجنسي؟5.

2.لا

1.الاسرة ب) إذاكانتالإجابةنعم،فماهومصدرالتربيةالجنسية؟

2.الاصدقاء

3.مدرسة

4.الاطباء/عاملالصحة

5.غيرذالك (حدد)_________________

**المعرفةعلىوسائلمنعالحمل**

1.نعم أ) هلسبقأنسمعتعنوسائلمنعالحملالحديثة؟1.

2.لا

1.الاسرة ب) إذاكانتالإجابةبنعم،منأينأومنسمعتعنها؟

2. الأصدقاء

3.المدرسة

4. عاملالصحة.

5. مجموعةالدراما

6.الاعلانات

7. غيرذلك (حدد) __________

1.نعم ج) هلتعرفاثنينمنوسائلمنعالحملالحديثة؟

2. لا

1. الأزواجالمتزوجينفقط منبرأيكيجباستخداموسائلمنعالحملالحديثة؟2.

2. الناسالنشطين

3. بالغينفقط

4. أيشخصيريد

5. لاأعلم

1. تباعدالأطفال ماالذييجبأنتستخدمهموانعالحمل؟3.

2. الحدمنالأطفال

3. في 1 و 2

4. يجبألاتستخدمعلىالإطلاق

ماهوالدورالذيتعتقدأنهللحصولعليهوقبولهواكتسابالمعرفةحولاستخداموسائلمنعالحمل؟4.

1. الرجالفقط

2. المرأةفقط

3.كلاهما

1.نعم هلسبقلكاستخدامأيوسائلمنعالحملالحديثة؟1.

2.لا

1.نعم هلتستخدمحالياأيوسائلمنعالحملالحديثة؟2.

2.لا

  ● إذاكانتالإجابةبنعم،أجب (3) و (4). إذاكانالجوابلا،أجب (6).

1.كوندوم ماهيوسيلةمنعالحملالحديثةالتيتستخدمهاحاليًا؟3.

2. مانعالحمل (حبوبمنعالحمل

3. مانعللحقنمانعللحقنة

4.زرع

5.اللوب

6 - غيرذلك (يرجىالتحديد) ________

_________ سنوات / ______ شهور منذمتىوأنتتستخدموسيلةمنعالحملالحديثةالمذكورةفي (3) أعلاه؟4.

1. غيرنشطةجنسيا أسبابعدماستخداموسائلمنعالحملالحديثة5.

2. المحظوراتالمروعة

3. المحظوراتالثقافية

4. محظوراتالشريك

5. أسبابصحية

6. الخوفمنالآثارالجانبية

7. نقصالمعرفة

8. أخرى (حدد) ___________

1.نعم هلتفكرفياستخدامأيشكلمنأشكالوسائلمنعالحملالحديثةفيالمستقبل؟6.

2.لا

1. الواقيالذكري إذاكانتالإجابةبنعم،فماهوالشيءالذيسوفتضعهفياعتبارك؟7.

2. مانعالحمل (حبوبمنعالحمل)

3. وسائلمنعالحملالحمية

4.زرع

5.اللوب

6. أخرى (حدد) ___________

1. مرفقصحي ماهومصدر (مصادر) وسائلمنعالحملالحديثةالتيتعرفها؟8.

2.الاصدقاء

3. الأسرة

4. أخرى (حدد) __________

1.نعم هلتعرفمصدرمانعللحملفي 10 دقائق؟9.

2.لا

شكرالمشاركتك

**APPENDIX XIII: QUALITATIVE INTERVIEW GUIDE**

**LIVED EXPERIENCES OF REFUGEE ADOLESCENTS ON MODERN CONTRACEPTIVES IN NORTHERN UGANDA**

**INTRODUCTION (***Introduce yourself, shake hands with interviewee*)

Thank you for sparing your time to participate in this interview. The purpose of this interview is to understand the lived experiences of refugee adolescents on modern contraceptives in Northern Uganda, and the information obtained from this interview will guide us in knowing how best we can help adolescents using modern contraceptives to adhere and comply to them, which in the long run will help reduce adolescent pregnancies and their associated complications.

Participation in this study is very voluntary; you do not have to answer all the questions asked and you can stop the interview at any desired time. In order to ensure confidentiality, your responses will be kept anonymous by coding them, and the link between your name and the code will be kept in a secured and separate location.

I will start by asking you a few questions about yourself, and then move to your sexual and reproductive health history, continue to your experiences using the modern contraceptives, and finish with your recommendations.

This interview will take about 45mins to 1 hour.

“Do you have any questions before we continue?”

**Interviewer’s name: ........................................................... Sign: ..................................**

**Interviewee’s serial number: .............................................................................................**

**Start Time: ……………………………………… End Time: ………………….**

**Social Demographics**

1. Age:
2. Highest level of education:
3. Ethnicity:
4. Religion:
5. Occupation:
6. Marital status:
7. Years in the camp:

**Sexual and reproductive health**

1. Age at first sex:
2. Number of sexual partners:
3. Type of modern contraceptive used:
4. Duration of use of the contraceptive:

**Interview questions**

**A: Knowledge on modern contraceptives**

1. What comes to your mind when you hear about contraceptives?

*Probes: How did you know about the contraceptives?*

ii) What modern contraceptive methods do you know?

**B: Use of modern contraceptives**

1. What contraceptive method are you currently using or have you ever used?
2. For how long have you used or did you use the method?

*Probes: Why did you stop using it?*

**C: Access of modern contraceptives**

1. How do you get the contraceptives you use?

*Probes: Place, Distance, Cost, Time of access, Frequency, Challenges*

1. How easy is it to get the contraceptives when you need them?

*Probes: Availability, How long does it take at the service point*

**D: Contraceptive Support**

1. What information are you given while receiving the contraceptives?

*Probes: Instructions, Method advantages and disadvantages, Mode of action, Related side effects, How to stop use.*

1. In case of challenges, how do you deal with them?

*Probes: Who do you talk to, peers, health care providers. Do you feel free to talk about the challenges or you prefer to keep silent?*

1. What happens when society or peers find out that you are using contraceptives?

*Probes: Does anyone know?*

**E: Challenges faced**

1. What challenges have you faced while using the contraceptives?

*Probes: Access, Use, Side effects, Stigma, Services*

1. How did you manage them each of the challenges you faced?
2. How can the challenges you mentioned in (ii) be addressed in order to improve refugee adolescent contraceptive use?

Thank you for participating in this interview. The results of our study will be available to you. In appreciation of your time, we would like to give you a token of 10,000/=.

**APPENDIX XIV: QUALITATIVE INTERVIEW GUIDE (NUER TRANSLATION)**

**KACIE NYIRTHITƆKEKEYAABAKÖKKE YOK NE BIAK DE LUI DE KAKE GEL DE LIC NE LƆŊCIƐM DE UGANDA**

**Introduction/Gɔc de jäm (***luelrɔtkumɔthraanyenebathiecthok)*

Yin caleec ne thadun ca gamwuɔk ne yejameyic. Kekɔr bene yi thicthokɣebepiir de rïnythitɔ ne northern Uganda ɣekeyaabakökdetic le lɔŋ de lui de kake gel de dhithkunyiny bi yok ne thie bene yi thicthokabëwuɔnyuɔth te piɛthbewuɔkrïnythikuɔnythin ne lɔ̈ŋ de lui de kake gel dhithagoliny de nyirthitekickukɔkrɛckɔɔk.Tɔ du neyekeneyic a gamërɔt du ku yin le bathualcïkɔrpuɔlku yin le bathiccëthokrec ne thakɔr.

Acinkebegɔɔr bene yi nyic ne ka ca keluelku ka ca keluelebënabënyiɛctɔu. ɣɛnbethicgɔl ne thuelkebiak de rɔtdukujɔlyabiak de dhithkutɛɛckeneröragut ce kenyic ne biak de lui de kakedhithyickukebajaljaw ne keyic.

ɣejameabëtɔ ne kam de degɛkaikethierŋuankudhïcketha.

Nɔŋkekɔrbathickewuɔkenlɔtueŋ?

**Rinke raanthic k**ɔc**:..........................................................................**

**Thaany de ye cin:..................................**

**Namba de raanthicthok:..............................................................................................**

**Social Demographics/ kakechiɛŋ de ran**

1. Run:
2. Biak de thukul:
3. Jurdu:
4. Kanitha:
5. Luɔi:
6. Biak de thiik:
7. Run ne kamic:

**Sexual and reproductive health/biak de tɛɛc ke ror ku dhith**

1. Yin nɔŋ run kede ne yötueŋ du we moc:
2. Adeneror ye yöke yin
3. Kake gel de licluɔiɣekeek:
4. Thaca loi ke yin lui kake gel de lic

**Interview questions/thuɛl ke jamë**

1. Yekaŋo ca keyok ne lui de kake gel de licyic?
2. Yekaŋocie yi dhal ne lui de kakelicyic?
3. Yekacie yi dhal ne lui de kakelic(ii) yic leu wudë?
4. Yeyokbekeka ca keluelciedhal(ii) leu ɣadɛ̈ago luiden ne kam de rïnythicɔkpiny?

Yin caleec ne tɔ du neyejamëyic. Ka cukeyok ne yeluɔiɣeabëtɔ te kɔr yin keek. Yin lecku ne alïpkethiɛr ne thaduncagamwuɔk.

**APPENDIX XIV: QUALITATIVE INTERVIEW GUIDE (ARABIC TRANSLATION)**

التجارب المعيشية للمراهقين المعدمين على الموانع الحديثة في شمال أوغندا

دليل المقابلة

**المقدمة**

 (قدمنفسك،مصافحةمعمنأجريتالمقابلة)

شكراًلكعلىتداركوقتكللمشاركةفيهذهالمقابلة. الغرضمنهذهالمقابلةهوفهمالتجاربالمعيشيةللمراهقيناللاجئينعلىوسائلمنعالحملالحديثةفيشمالأوغندا،والمعلوماتالتيتمالحصولعليهامنهذهالمقابلةسوفترشدناإلىمعرفةأفضلالسبلالتييمكننابهامساعدةالمراهقينباستخداموسائلمنعالحملالحديثةللالتزاموالتقيدبها،علىالمدىالطويلسيساعدعلىتقليلحالاتالحملبينالمراهقاتوالمضاعفاتالمرتبطةبها.

المشاركةفيهذهالدراسةطوعيةللغاية. ليسعليكالإجابةعنجميعالأسئلةالمطروحةويمكنكإيقافالمقابلةفيأيوقتتريده. منأجلضمانالسرية،ستظلإجاباتكمجهولةمنخلالترميزها،وسيتمالاحتفاظبالارتباطبيناسمكوالرمزفيمكانآمنومنفصل.

سأبدأبسؤالبعضالأسئلةعننفسك،ثمانتقلإلىتاريخالصحةالجنسيةوالإنجابية،واستمرفيتجاربكباستخدامموانعالحملالحديثة،وانتهيبتوصياتك.

هذهالمقابلةتستغرقحوالي 45 دقيقةإلىساعةواحدة.

"هللديكأيأسئلةقبلأننستمر؟"

اسمالباحث:............................................... .................

  إشارة:..................................

الرقمالتسلسليللمقابلة: .............................................. ................................................

التركيبةالسكانيةالاجتماعية

أ) العمر:

ب) أعلىمستوىتعليمي:

ج) الهيئة:

د) الدين:

ه) المهنة:

و) الحالةالاجتماعية:

ز) سنواتفيالمعسكر:

الصحةالجنسيةوالإنجابية

أ) السنعندأولجنس:

ب) عددالشركاءالجنسيين:

ج) نوعموانعالحملالحديثةالمستخدمة:

د) مدةاستخدامموانعالحمل:

اسئلةالمقابلة

1) ماهيتجاربكباستخداموسائلمنعالحملالحديثة؟

2) ماهيبعضالتحدياتالتيواجهتهاأثناءاستخداموسائلمنعالحملالحديثة؟

3) كيفتمكنتمنإدارةكلمنالتحدياتالمذكورةفي (2) أعلاه؟

3) كيفيمكنمعالجةالتحدياتالتيذكرتهافي (2) منأجلتحسيناستخدامموانعالحمللدىالمراهقين؟

شكرالكعلىالمشاركةفيهذهالمقابلة. ستكوننتائجدراستنامتاحةلك. تقديرًالوقتك،نودأننمنحكرمزًاقدره 10000=.

**APPENDIX XVI**

**BUDGET**

| **ITEM** | **QUANTITY** | **DURATION** | **UNIT COST** | **UGX** | **USD** |
| --- | --- | --- | --- | --- | --- |
| **Research Ethics approval** |  |  |  |  |  |
| School of Med IRB | 1 |  | 1,800,000 | 1,800,000 | $486.49 |
| UNCST fees | 1 |  | 1,800,000 | 1,800,000 | $486.49 |
| Administrative clearance | 1 |  | 1,000,000 | 1,000,000 | $270.27 |
| **Sub-total** |  |  |  |  | **$1,243.24** |
| **Office admin costs** |  |  |  |  |  |
| Internet Expenses | 1 provider | 24 months | 50,000 | 1,200,000 | $324.32 |
| Communication expenses | 1provider | 24 months | 60,000 | 1,440,000 | $389.19 |
| Stationery and office supplies | 1 | 12 months | 250,000 | 3,000,000 | $810.81 |
| **Sub-total** |  |  |  |  | **$1,524.32** |
| **Capital items** |  |  |  |  |  |
| Laptop | 1 unit |  | 4,300,000 | 4,300,000 | $1,162.16 |
| Open digital software (qualitative | 1 unit |  | 1,500,000 | 1,500,000 | $405.41 |
| Quantitative software) | 1 unit |  | 1,500,000 | 1,500,000 | $405.41 |
| External drive | 2 units |  | 250,000 | 500,000 | $135.14 |
| **Sub-total** |  |  |  |  | **$2,108.11** |
| **Trainings and Conferences for the PI** |  |  |  |  |  |
| 2 international conferences | 2 travel expenses |  | 9,000,000 | 18,000,000 | $4,864.86 |
| **Sub-total** |  |  |  |  | **$4,864.86** |
| **Team Training** |  |  |  |  |  |
| ***Team Training for Qualitative data and reviewing tools*** |  |  |  |  |  |
| Refreshments | 8 persons | 2 days | 240,000 | 480,000 | $129.73 |
| Logistical costs | 1 training | 2 days | 100,000 | 200,000 | $54.05 |
| ***Team Training for Contraceptive methods*** |  |  |  |  |  |
| Refreshments | 8 persons | 2 days | 240,000 | 480,000 | $129.73 |
| Logistical costs | 1 training | 2 days | 100,000 | 200,000 | $54.05 |
| **Sub-total** |  |  |  |  | **$367.57** |
| **Pre-visit to the study site** |  |  |  |  |  |
| Transport costs to and from Kampala to Adjumani | 1 |  | 512,000 | 512,000 | $138.38 |
| Local travel | 1 |  | 30,000 | 30,000 | $8.11 |
| Car hire | 1 car | 5 days | 150,000 | 750,000 | $202.70 |
| Per Diem | 1 | 5days | 140,000 | 700,000 | $189.19 |
| **Sub-total** |  |  |  |  | **$538.38** |
| **Data collection** |  |  |  |  |  |
| ***Study I data collection*** |  |  |  |  |  |
| Transport costs to and from Adjumani | 1 |  | 512,000 | 512,000 | $138.38 |
| Per diem costs | 1 | 60 days | 140,000 | 8,400,000 | $2,270.27 |
| Research Assistants | 2 | 3 months | 500,000 | 3,000,000 | $810.81 |
| Participants’ compensation | 835 Participants |  | 5,000 | 4,175,000 | $1,128.38 |
| **Sub-total** |  |  |  |  | **$4,347.46** |
| ***Study II data collection*** |  |  |  |  |  |
| Transport costs to and from Adjumani | 1 |  | 512,000 | 512,000 | $138.38 |
| Per diem costs | 1 | 90 days | 140,000 | 12,600,000 | $3,405.41 |
| Research Assistants | 2 | 3 months | 500,000 | 3,000,000 | $810.81 |
| Participants’ compensation | 588 participants |  | 5,000 | 2,940,000 | $794.59 |
| Peer counsellors | 5 | 3 months | 300,000 | 4,500,000 | $1,216.22 |
| Nurse counsellors | 3 | 3 months | 500,000 | 4,500,000 | $1,216.22 |
| Male condoms | 100 boxes |  | 10,000 | 1,000,000 | $270.27 |
| Female condoms | 20 boxes |  | 20,000 | 400,000 | $108.11 |
| Implants | 100 packs |  | 10,000 | 1,000,000 | $270.27 |
| Injectable contraceptives | 100 packs |  | 5,000 | 500,000 | $135.14 |
| IUDs | 50 packs |  | 20,000 | 1,000,000 | $270.27 |
| Alcohol Disinfectant and hand rub | 10 1 litre bottles |  | 30,000 | 300,000 | $81.08 |
| Disposable gloves | 20 boxes |  | 20,000 | 400,000 | $108.11 |
| Syringes | 10 boxes |  | 20,000 | 200,000 | $54.05 |
| Cotton wool | 5 rolls |  | 20,000 | 100,000 | $27.03 |
| Alcohol swabs | 10 packets |  | 10,000 | 100,000 | $27.03 |
|  |  |  |  |  |  |
| **Sub-total** |  |  |  |  | **$8,932.97** |
| ***Study III Data collection*** |  |  |  |  |  |
| Transport costs to and from Adjumani | 1 | 1 | 512,000 | 512,000 | $138.38 |
| Per diem costs | 1 | 180 days | 140,000 | 25,200,000 | $6,810.81 |
| Research Assistants | 2 | 6 months | 500,000 | 6,000,000 | $1,621.62 |
| Participants’ compensation | 385 participants |  | 5,000 | 1,925,000 | $520.27 |
| **Sub-total** |  |  |  |  | **$9,091.08** |
| ***Study IV Data collection*** |  |  |  |  |  |
| Transport costs to and from Adjumani | 1 |  | 512,000 | 512,000 | $138.38 |
| Per Diem | 1 | 90 days | 140,000 | 12,600,000 | $3,405.41 |
| Research assistants | 2 | 3 months | 500,000 | 3,000,000 | $810.81 |
| Recorders | 2 |  | 500,000 | 1,000,000 | $270.27 |
| Participant compensation | 1 | 50 | 10,000 | 500,000 | $135.14 |
| **Sub-total** |  |  |  |  | **$4,760.00** |
| **Data management** |  |  |  |  |  |
| Quantitative data entry | 1735 Questionnaires |  | 5,000 | 8,675,000 | $2,344.59 |
| Qualitative data transcription | 20 sets |  | 50,000 | 1,000,000 | $270.27 |
| Consultation for analysis |  |  | 1,000,000 | 1,000,000 | $270.27 |
| ***Dissemination and publication*** |  |  |  |  |  |
| Dissemination Seminar | 25 |  |  | 3,600,000 | $972.97 |
| Publication fees | 2 Publications |  | @ $500 | 3,700,000 | $1,000.00 |
| **Sub-total** |  |  |  |  | **$4,857.11** |
| **Principal investigator** |  |  |  |  |  |
| Principal investigator stipend | 1 | 24 months | 1,800,000 | 43,200,000 | $11,675.68 |
| **GRAND TOTAL** |  |  |  | **200,590,024** | **$54,213.52** |

**BUDGET JUSTIFICATION**

**Personnel**

1. Principal Investigator: the principal investigator will be responsible for the preparation, conduct and administration of the research, in addition to co-ordinating and supervising all the research activities. This will involve hiring, training and mentoring of the research team in all study activities and ethical conduct of the research participants, participant recruitment, supervising the interview processes, preparing staff progress meetings, data management, analysis, and reporting and dissemination of study findings. This is planned for a period of 24 months.
2. Research Assistants: The research assistants will help with the data collection, both quantitative and qualitative, particularly through conducting interviews. Research assistants will also help to take the research participants through the consent process before they can do the interviewing. These will also be in charge of administering modern contraceptives to participants who may need them.
3. Peer counsellors: These will be any five female adolescents selected and trained in contraceptive counselling so as to offer this counselling to their fellow peers. This will be the intervention in study II. Peer counsellors will work for the duration of the study II which is anticipated to last 3 months.
4. Nurse counsellors: These will be any two nurses selected from the community health centres who will offer contraceptive counselling to a selected group of research participants as the control of the study in study II. Nurse counsellors will work for a period of 3 months, the duration of study II.
5. Data entrants: These will be any individuals selected for the purpose of entering data from questionnaires into Epidata software and for ensuring proper data management.
6. Analysis consultants: Statisticians will be consulted regarding the analysis of the quantitative data for all the three quantitative studies.

**Other direct costs**

1. Travel costs: Distance from Kampala to Adjumani is 445Km, and a litre of fuel is expected to cover a distance of 7Km. Fuel needed to drive to Adjumani is (445/7) 64 litres of fuel. Each litre of fuel costs 4,000 Ush, therefore (4,000*64*2) 512,000 Ush is the amount needed to travel to and from Adjumani.
2. Research consumables: This will include modern contraceptives like the condoms (both male and female), oral contraceptives, injectables, implants and the intra-uterine devices (IUDs) which will be provided to the participants on decision to use modern contraceptives.
3. Other consumables such as alcohol swabs, gloves, cotton wool and syringes will be used in the administration of modern contraceptives like the implants, IUDs and injectables to participants who will have chosen them.
4. Participant costs: Study participants will be reimbursed a transport cost or compensation for time of 5,000/= for studies 1, II and III, and 10,000/= for study IV because it requires longer interviews as required by the Uganda National Council of Science and Technology.

# APPENDIX XVII

# ACTIVITY PLAN

| **Activities** | M | J | J | A | S | O | N | D | J | F | M | A | M | J | J | A | S | O | N | D | J | F | M | A | M | J |
| --- | --- | --- | --- | --- | --- | --- | --- | --- | --- | --- | --- | --- | --- | --- | --- | --- | --- | --- | --- | --- | --- | --- | --- | --- | --- | --- |
| Proposal writing |  |  |  |  |  |  |  |  |  |  |  |  |  |  |  |  |  |  |  |  |  |  |  |  |  |  |
| Study site visit |  |  |  |  |  |  |  |  |  |  |  |  |  |  |  |  |  |  |  |  |  |  |  |  |  |  |
| Proposal submission  n and approvals |  |  |  |  |  |  |  |  |  |  |  |  |  |  |  |  |  |  |  |  |  |  |  |  |  |  |
| Planning meetings |  |  |  |  |  |  |  |  |  |  |  |  |  |  |  |  |  |  |  |  |  |  |  |  |  |  |
| Data collection (study I) |  |  |  |  |  |  |  |  |  |  |  |  |  |  |  |  |  |  |  |  |  |  |  |  |  |  |
| Attend cross cutting courses |  |  |  |  |  |  |  |  |  |  |  |  |  |  |  |  |  |  |  |  |  |  |  |  |  |  |
| Study I data analysis |  |  |  |  |  |  |  |  |  |  |  |  |  |  |  |  |  |  |  |  |  |  |  |  |  |  |
| Data collection (study II) |  |  |  |  |  |  |  |  |  |  |  |  |  |  |  |  |  |  |  |  |  |  |  |  |  |  |
| Study II Data Analysis |  |  |  |  |  |  |  |  |  |  |  |  |  |  |  |  |  |  |  |  |  |  |  |  |  |  |
| Data collection (study III) |  |  |  |  |  |  |  |  |  |  |  |  |  |  |  |  |  |  |  |  |  |  |  |  |  |  |
| Publication I |  |  |  |  |  |  |  |  |  |  |  |  |  |  |  |  |  |  |  |  |  |  |  |  |  |  |
| Study III follow up at 1 month |  |  |  |  |  |  |  |  |  |  |  |  |  |  |  |  |  |  |  |  |  |  |  |  |  |  |
| Study III follow up at 3 months |  |  |  |  |  |  |  |  |  |  |  |  |  |  |  |  |  |  |  |  |  |  |  |  |  |  |
| Attend cross cutting courses |  |  |  |  |  |  |  |  |  |  |  |  |  |  |  |  |  |  |  |  |  |  |  |  |  |  |
| Study III follow up at 6  Months |  |  |  |  |  |  |  |  |  |  |  |  |  |  |  |  |  |  |  |  |  |  |  |  |  |  |
| Data collection (Study IV) |  |  |  |  |  |  |  |  |  |  |  |  |  |  |  |  |  |  |  |  |  |  |  |  |  |  |
| Publication II |  |  |  |  |  |  |  |  |  |  |  |  |  |  |  |  |  |  |  |  |  |  |  |  |  |  |
| Study III analysis |  |  |  |  |  |  |  |  |  |  |  |  |  |  |  |  |  |  |  |  |  |  |  |  |  |  |
| Study IV analysis |  |  |  |  |  |  |  |  |  |  |  |  |  |  |  |  |  |  |  |  |  |  |  |  |  |  |
| Dissertation writing |  |  |  |  |  |  |  |  |  |  |  |  |  |  |  |  |  |  |  |  |  |  |  |  |  |  |
| Defense |  |  |  |  |  |  |  |  |  |  |  |  |  |  |  |  |  |  |  |  |  |  |  |  |  |  |
